# Modelling G protein-biased agonism using GLP-1 receptor C-terminal mutations

**DOI:** 10.1016/j.molmet.2026.102321

**Published:** 2026-01-20

**Authors:** Hanh Duyen Tran, Yiming Zuo, Carissa Wong, Alice Pollard, Steve Bloom, Ben Jones

**Affiliations:** 1 Section of Endocrinology, Department of Metabolism, Digestion and Reproduction, Faculty of Medicine, Imperial College London, Du Cane Road, W12 0NN, United Kingdom; 2 Institute of Clinical Sciences, Faculty of Medicine, Imperial College London, Du Cane Road, W12 0NN, United Kingdom

**Keywords:** GLP-1 receptor, β-arrestin, Biased agonism

## Abstract

**Background and aim:**

The glucagon-like peptide-1 receptor (GLP-1R) is a major therapeutic target for type 2 diabetes and obesity. Agonists showing bias in favour of G protein signalling over β-arrestin recruitment and GLP-1R internalisation, e.g. tirzepatide and orforglipron, have favourable clinical efficacy profiles. However, understanding of the effects of biased agonism has been hampered by differences in ligand properties such as affinity, efficacy, stability and pharmacokinetics. Here we used GLP-1R C-tail mutations that inhibit phosphorylation to mimic G protein-biased GLP-1R agonism without the need for ligand modifications.

**Methods:**

Serine doublet phosphorylation sites in the human and mouse GLP-1R C-tails were mutated to alanine. Wild-type and mutant GLP-1Rs were examined for β-arrestin recruitment, internalisation, Gα_s_ activation, and signalling readouts in HEK293 cells and pancreatic β-cell models. Native GLP-1 plus oppositely biased ligands exendin-phe1 (ExF1; G protein-biased) and exendin-asp3 (ExD3; β-arrestin-biased) were used to compare ligand- and receptor-mediated biased agonism.

**Results:**

Loss of three C-terminal phosphorylation sites reduced GLP-1- and ExD3-mediated GLP-1R internalisation and β-arrestin recruitment to that seen with ExF1. The phosphodeficient GLP-1R showed preferential plasma membrane Gα_s_ activation over longer stimulations, with associated increases in whole cell cAMP generation and kinomic signalling. The distal GLP-1R phosphorylation site played a larger role in β-arrestin recruitment, and the proximal sites were more important for GLP-1R internalisation and regulating cAMP production.

**Conclusions:**

Genetic changes that reduce β-arrestin recruitment and slow GLP-1R internalisation can enhance GLP-1R signalling, providing conceptual support for the use of G protein bias to improve GLP-1R agonist efficacy.

## Highlights

• Mutating GLP-1R C-terminal phosphorylation sites mimics G protein-biased agonism.
• Phosphorylation sites modulate nature and subcellular location of GLP-1R signalling.
• Distinct roles of different phosphorylation sites on internalisation *versus* β-arrestin recruitment.

## 1 Introduction

Glucagon-like peptide-1 receptor agonists (GLP-1RAs) are a major treatment class for type 2 diabetes and obesity. Mimicking the action of the native GLP-1 hormone, GLP-1RAs reduce blood glucose by enhancing insulin release from pancreatic β-cells and slowing the intestinal transit and subsequent absorption of ingested glucose. They also produce substantial weight loss by activating appetite-regulating neurons in the brain [1].

Several GLP-1RAs are now approved for clinical use. The most recently approved example, tirzepatide, shows two distinct pharmacological properties that distinguish it from earlier agents such as exenatide, liraglutide, dulaglutide and semaglutide. Firstly, tirzepatide is a dual agonist ligand which jointly targets the glucose-dependent insulinotropic polypeptide receptor (GIPR) as well as GLP-1R [2]. Secondly, at the GLP-1R, it has a distinct signalling profile characterised by markedly reduced recruitment of β-arrestins to the receptor and slower internalisation, but retained ability to activate the Gα_s_/adenylate cyclase/cAMP pathway [3]. The former two processes, best known for their role in terminating receptor signalling, are important for fine temporal regulation of physiological responses, but might limit achievable pharmacotherapeutic efficacy during sustained stimulations. Therefore, avoiding β-arrestin recruitment and GLP-1R internalisation via “G protein-biased” agonism may be a way to reduce the tendency for ligand responses to decline over time [4]. Orforglipron, a non-peptide GLP-1RA designed for oral administration, and the long-acting peptide ecnoglutide, also show G protein bias at the GLP-1R and have shown positive effects in clinical trials [[5], [6], [7], [8]].

However, defining the role of G protein-biased agonism in GLP-1R signalling remains challenging. Whilst β-arrestins are classically known for GPCR desensitisation via steric hindrance of G protein binding, they can also drive G protein-independent signalling e.g. via ERK1/2. Moreover, whilst their canonical role in coupling to endocytic proteins to drive receptor internalisation eventually leads to receptor downregulation, it also contributes to signalling by delivering active GPCR complexes to signalling endosomes. The potential for β-arrestins and endocytosis to both positively and negatively regulate ligand responses is reflected in the GLP-1R literature: genetic depletion studies show that β-arrestins contribute to insulin secretion, cytoprotection and acute *in vivo* glucoregulatory responses to GLP-1RAs [[9], [10], [11]], whilst many other studies show improved sustained anti-hyperglycaemic and weight loss effects when ligand-induced β-arrestin recruitment and/or GLP-1R internalisation are avoided [[11], [12], [13], [14], [15], [16], [17], [18], [19]]. Of note, both ligand and standard genetic approaches to achieve biased agonism carry certain caveats. Firstly, interpretation of biased agonist studies can be confounded by differences in ligand affinity, partial agonism or pharmacokinetics [3,15,16]. Secondly, functional redundancy between β-arrestin-1 and -2 means both isoforms need to be deleted to produce clear cut effects, but this can produce extensive “rewiring” and cellular abnormalities [20]. Moreover, several reports have confirmed that GLP-1R endocytosis does not require β-arrestins [13,[21], [22], [23], [24], [25]], posing the question of which of reduced internalisation and reduced β-arrestin recruitment primarily drives the advantageous properties of G protein-biased GLP-1R agonists.

An alternative genetic approach to model biased agonism, which we advance in this study, is to introduce receptor modifications that selectively modulate transducer coupling. Correlation between genomic variants in drug targets and phenotype have frequently been used as proxies for drug actions [[26], [27], [28]]. Mutagenesis studies have revealed numerous residues linking GLP-1R activation to β-arrestin recruitment and/or β-arrestin-dependent downstream signalling, for example in the extracellular surface of the receptor [29,30]. However, when targeting these β-arrestin-selective sites, it is difficult to completely avoid alterations in other pathways. Instead, here we evaluate the effect of disrupting receptor C-terminal serine phosphorylation, which is typically required to produce high affinity β-arrestin recruitment. A prescient study from 1997 highlighted three serine doublets (S441/442, S444/445 and S451/452) as key phosphorylation sites in the rat GLP-1R C-terminus which, when mutated to alanine, reduced the uptake of radiolabelled GLP-1 (used as a proxy for receptor internalisation) and reduced GLP-1R desensitisation [31]. The authors predicted a role for β-arrestins but, due to technological limitations, this was not measured directly. Reduced recruitment of β-arrestin to GLP-1R with several serine/threonine C-terminal mutations has since been confirmed [32,33].

To build on this earlier work, we perform an updated characterisation of the SS→AA mutations equivalent to those studied in the rat GLP-1R by Widmann et al. [31], aiming to 1) measure β-arrestin-1 and -2 recruitment and receptor internalisation directly, 2) determine the impact on subcellular location of transducer coupling, and 3) establish the functional consequences of these differences on downstream processes including wider signalling readouts and insulin secretion from pancreatic β-cells. Human and mouse GLP-1R were included as mice are frequently used in GLP-1R research, so awareness of similarities and differences is important for translation. To compare ligand and receptor-driven biased agonism we included the oppositely biased GLP-1RAs exendin-asp3 (ExD3) and exendin-phe1 (ExF1) which, respectively, enhance or avoid β-arrestin and internalisation responses [13], and are frequently used as tools to investigate GLP-1R biased agonism [15,23,[34], [35], [36], [37], [38], [39]]. Hence, our study provides a ligand-independent view of the cellular consequences of G protein-biased GLP-1R agonism.

## 2 Results

### 2.1 Loss of three putative human GLP-1R C-terminal phosphorylation sites reduces β-arrestin-2 recruitment and activation, with an even larger effect on β-arrestin-1

**Figure 1:**
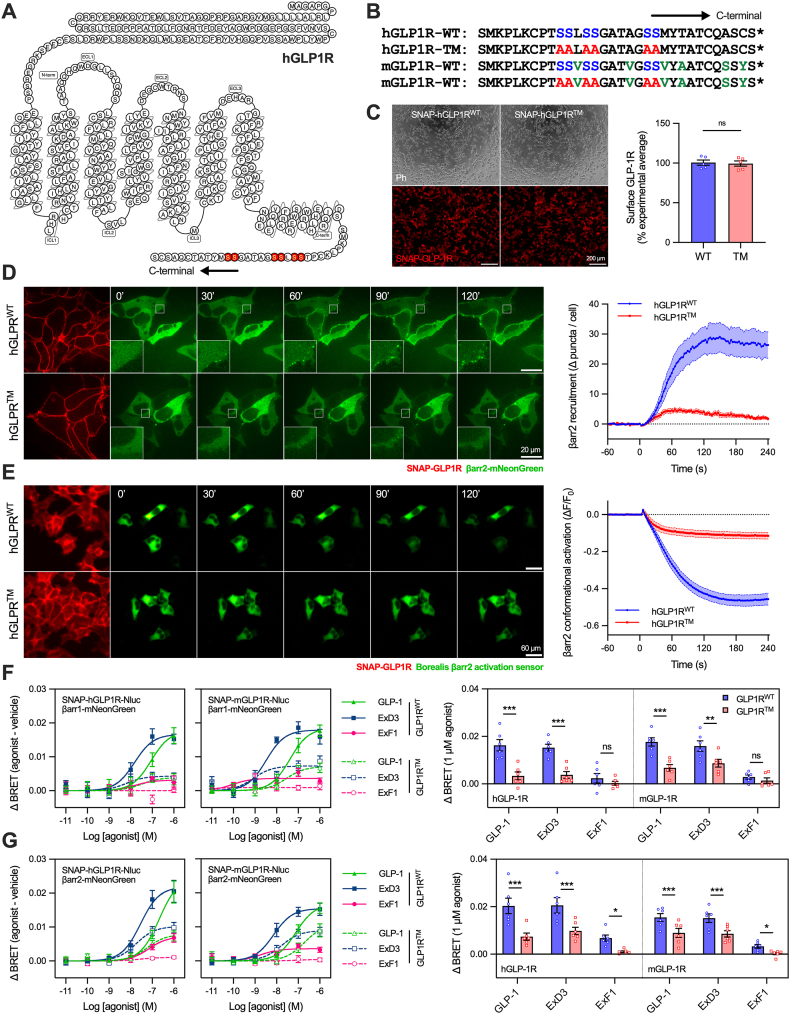
**Impaired β-arrestin responses with the triple phosphomutant GLP-1R.** (**A**) hGLP-1R sequence, with three putative phosphorylation sites highlighted in red. (**B**) Sequence alignment of hGLP1R and mGLP1R C-termini indicating the sequence of the corresponding “triple mutants”. (**C**) Representative images and surface expression quantification of stable SNAP-hGLP1R^WT^ and SNAP-hGLP1R^TM^ cells, *n* = 5, paired t-test. Scale bar = 200 μm. (**D**) Time-lapse confocal images of β-arrestin-2-mNeonGreen recruitment (scale bar = 20 μm) and quantification from *n* = 5 repeats. (**E**) Time-lapse epifluorescence images of β-arrestin-2 Borealis conformational activation (indicated by a reduction in fluorescence intensity; scale bar = 60 μm) and quantification from *n* = 5 repeats. (**F**) β-arrestin-1-mNeonGreen recruitment to SNAP-GLP-1R-Nluc in transiently transfected AD293 cells treated for 5 min with GLP-1, ExD3 or ExF1, *n* = 6, with two-way matched ANOVA with Šidák's test comparing response at 1 μM. (**G**) As for (F) but with β-arrestin-2-mNeonGreen. ∗p < 0.05, ∗∗p < 0.01, ∗∗∗p < 0.001 by indicated statistical test. All data shown as mean ± SEM, with individual replicates in some cases.

Based on the three C-terminal serine doublets identified as phosphorylation sites in the rat GLP-1R [31], we obtained constructs encoding the human GLP-1R with and without the corresponding alanine mutations at the same three sites. We refer to these as hGLP1R^WT^ for wild-type and hGLP1R^TM^ for “triple mutant”, respectively (Fig. 1A, B). N-terminal SNAP-tags were included to facilitate receptor visualisation. Stable HEK293-derived Flp-In cells with homogenous expression of each receptor were generated, with surface-specific SNAP-labelling confirming similar plasma membrane receptor densities (Fig. 1C). Live cell imaging of SNAP-hGLP1R^WT^ cells showed rapid translocation to the plasma membrane of β-arrestin-2 tagged with the bright green fluorescent protein (GFP) variant mNeonGreen [40] within 30 seconds of application of a saturating concentration (1 μM) of GLP-1, where it appeared in punctate structures (Fig. 1D, Supplementary Video 1). SNAP-hGLP1R^TM^ cells showed much reduced but still detectable β-arrestin recruitment. As β-arrestins need not only to be recruited but also activated to fulfil some of their functions [41], we also recorded responses using the Borealis β-arrestin-2 activation sensor, which includes a circularly permuted GFP variant tag designed to produce a decrease in fluorescence intensity when the β-arrestin undergoes conformational rearrangement [42]. This experiment confirmed activation and recruitment of β-arrestin-2 show similar kinetics (Fig. 1E). Moreover, the β-arrestin-preferring full agonist ExD3 produced robust recruitment of β-arrestin-2 in SNAP-hGLP1R^WT^ cells but far less in SNAP-hGLP1R^TM^ cells, whereas the β-arrestin-avoiding partial agonist ExF1 led to very little recruitment with either receptor (Supplementary Fig. 1A). Impaired recruitment of β-arrestin-1 with the triple mutant receptor was even more marked than for β-arrestin-2 (Supplementary Fig. 1A).

Supplementary video related to this article can be found at https://doi.org/10.1016/j.molmet.2026.102321

The following is/are the supplementary data related to this article:

Supplementary Video 1 1 Supplementary Video 1

These findings were supported by BRET measurements using C-terminal nanoluciferase tagged SNAP-hGLP1R transiently transfected in adherent HEK293 (AD293) cells. β-arrestin-1 and -2 recruitment at 5 min were substantially reduced in response to ExD3 and GLP-1 at the triple mutant receptor (Fig. 1F, G), with numerical reductions in the small amount of ExF1-induced recruitment. The mutation effect was larger with β-arrestin-1 compared to β-arrestin-2 (∼80% *versus* ∼50% reduction in ExD3-induced recruitment, respectively). For the equivalent mouse GLP-1R constructs (see Fig. 1B), referred to as mGLP1R^WT^ and mGLP1R^TM^, the pattern was similar to with the human receptors, except that the triple mutation reduced β-arrestin-1 and -2 recruitment to a similar extent (Fig. 1F, G). The mGLP1R^TM^ construct showed moderately lower surface expression than the equivalent wild-type receptor (Supplementary Fig 1B); however, this is unlikely to materially affect the BRET assay results, in which responses are expressed ratiometrically. The phosphodeficient mutants affected the maximum responses rather than potencies (Supplementary Fig 1C). Acute conformational activation of β-arrestin-2 in response to 1 μM ExD3 or GLP-1 was markedly reduced at the triple mutant receptor for both species (tested this time without the C-terminal luciferase), with the small amount of ExF1-induced β-arrestin-2 activation also abolished (Supplementary Fig. 1D and 1E).

These data confirm that the three C-terminal serine doublets mutated in these experiments are necessary to drive full recruitment and activation of β-arrestin to the human and mouse GLP-1R. It is notable that the amount of residual β-arrestin recruitment with the mutant hGLP-1R in response to GLP-1 or ExD3 was similar to that achieved with ExF1 at the wild-type hGLP-1R, suggesting that inhibiting GLP-1R C-tail phosphorylation may be a way to mimic G protein-biased agonism.

### 2.2 Loss of three phosphorylation sites reduces GLP-1R internalisation

**Figure 2:**
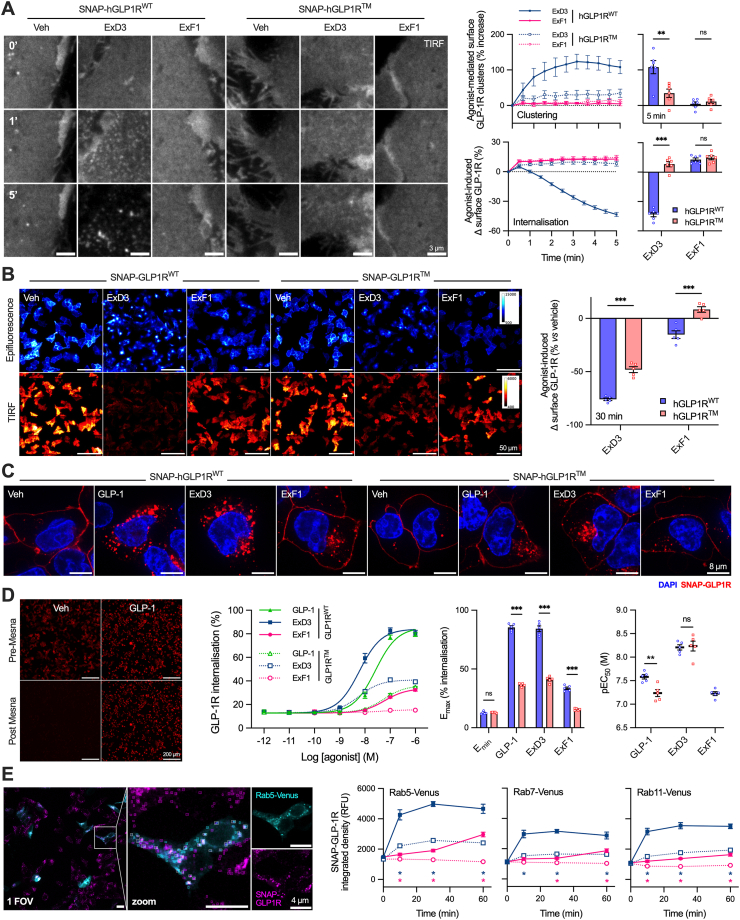
**Impaired internalisation responses with the triple phosphomutant GLP-1R.** (**A**) Representative TIRF images showing stable SNAP-hGLP1R^WT^ or SNAP-hGLP1R^TM^ cells treated with 1 μM agonist, scale bar = 3 μm. Appearance of GLP-1R clusters and disappearance from the plasma membrane is most apparent in the hGLP1R^WT^ + ExD3 images. The graphs show quantification of GLP-1R clusters and internalisation, *n* = 6, with two-way matched ANOVA with Šidák's test comparing data at 5 min. (**B**) Quantification of loss of surface GLP-1 in stable SNAP-hGLP1R^WT^ or SNAP-hGLP1R^TM^ cells treated with 1 μM agonist for 30 min, scale bar = 50 μm; loss of cell surface GLP-1R is quantified the TIRF:epifluorescence ratio and then as a percentage change from vehicle treated cells; comparison by two-way matched ANOVA with Šidák's test from *n* = 5 experiments. (**C**) Representative images showing stable SNAP-hGLP1R^WT^ or SNAP-hGLP1R^TM^ cells 30 min after stimulation with vehicle or 1 μM agonist, scale bar = 8 μm. (**D**) GLP-1R internalisation at 30 min in stable SNAP-hGLP1R^WT^ or SNAP-hGLP1R^TM^ cells quantified by reversible SNAP-GLP1R labelling, *n* = 5, with comparisons between E_max_ and pEC_50_ by two-way matched ANOVA with Šidák's test. (**E**) Representative image from high content colocalisation analysis of stable SNAP-hGLP1R^WT^ or SNAP-hGLP1R^TM^ cells transiently transfected with Rab-Venus constructs and treated with 1 μM ExD3 for 30 min, scale bar = 4 μm. The inset indicates shows identified objects in each channel, with colocalised objects in white. Statistical comparison from *n* = 5 experiments by two-way matched ANOVA with Sidak's test, with the asterisk colour highlighting genotype-specific differences for each agonist at each time-point. ∗p < 0.05, ∗∗p < 0.01, ∗∗∗p < 0.001, by indicated statistical test. All data shown as mean ± SEM, with individual replicates in some cases.

It was reported that cells expressing phosphorylation-deficient rat GLP-1R showed impaired uptake of radiolabelled GLP-1 [31], suggesting impaired GLP-1R internalisation. Imaging the cell surface of stable SNAP-hGLP1R^WT^ cells by total internal reflectance fluorescence (TIRF) microscopy, we observed rapid formation of GLP-1R clusters when treated with ExD3, shortly followed by progressive disappearance of receptor from the cell surface (Fig. 2A). Cluster formation in hGLP1R^TM^ cells was much less prominent but still detectable with ExD3, and there was little evidence of GLP-1R internalisation over the short 5-minute incubation. ExF1 responses were barely distinguishable from vehicle, except for a paradoxical increase in TIRF signal at the first time-point seen with both agonists, likely to be related to changes in cell morphology due to receptor activation. Higher throughput combined TIRF and epifluorescence imaging of fixed cells after a 30-minute stimulation showed extensive loss of surface hGLP1R^WT^ with ExD3, with this response approximately halved with hGLP1R^TM^ (Fig. 2B). Of note, ExF1 induced a moderate amount of hGLP1R^WT^ internalisation but none of hGLP1R^TM^; this suggests that GLP-1R endocytosis with ExF1, although of lower magnitude, also requires C-tail phosphorylation. Confocal imaging corroborated these findings by demonstrating accumulation of SNAP-GLP-1R inside the cell, with native GLP-1 showing a similar pattern to ExD3 (Fig. 2C). GLP-1R internalisation was quantified using a high throughput reversible labelling assay in which a cleavable SNAP-tag probe (BG–SS–AF647) is used to label surface SNAP-GLP1R, followed by agonist treatment and imaging before and after the reducing agent Mesna to remove surface fluorescence (Fig. 2D). This assay again confirmed reductions in GLP-1R internalisation with hGLP1R^TM^ with all ligands. ExD3 showed the greatest resistance to the effects of the mutation, with ∼40% of maximal agonist-induced internalisation preserved and potency unaltered, compared to GLP-1 which for which the mutation led to reduced potency and a slightly larger loss in maximal internalisation.

Monitoring formation of endosomal puncta by widefield imaging, it was apparent that hGLP1R^WT^ produces rapid internalisation with GLP-1 and ExD3, whereas at hGLP1R^TM^ the response was both slower and of reduced magnitude (Supplementary Fig. 2A). To characterise post-endocytic trafficking, we used high content confocal imaging to measure colocalisation of internalised SNAP-GLP-1R with early, late and recycling endosomes in hundreds of cells by co-transfecting Rab5-Venus, Rab7-Venus or Rab11-Venus, respectively (Fig. 2E). These experiments highlighted rapid ExD3-induced distribution of hGLP1R^WT^ to all measured endosomal compartments, in keeping with previous results [16,43]. Substantially less endosomal localisation of hGLP1R^TM^ was seen with both ExD3 and ExF1. Although displaying a somewhat different kinetic pattern, after sustained stimulation the ExD3/hGLP1R^TM^ and ExF1/hGLP1R^WT^ combinations produced a reasonably similar subcellular distribution of internalised GLP-1R.

Additional data were collected to compare human and mouse GLP-1R responses. Agonist-induced decreases in BRET between GLP-1R-Nluc and the plasma membrane marker KRAS-Venus, indicating disappearance of the receptor from the cell surface, showed the expected pattern: all responses were significantly slowed by the triple phosphosite mutations at both GLP-1R species (Supplementary Fig. 2B). Similar effects of the phosphorylation deficient mutations were apparent from confocal imaging of transiently transfected cells (Supplementary Fig. 2C), and quantification of internalisation using the reversible labelling assay showed similar responses to those seen with the stable hGLP-1R cell lines (Supplementary Fig. 2D).

These data demonstrate that GLP-1R C-tail phosphosites are needed for full agonist-induced GLP-1R internalisation, although a lesser amount is still possible when they are disrupted. The internalisation pattern seen with ExD3 at the phosphorylation-deficient GLP-1R was a reasonable approximation of that seen with ExF1 at the wild-type GLP-1R.

### 2.3 Loss of three GLP-1R phosphorylation sites alters the subcellular localisation of β-arrestin recruitment

**Figure 3:**
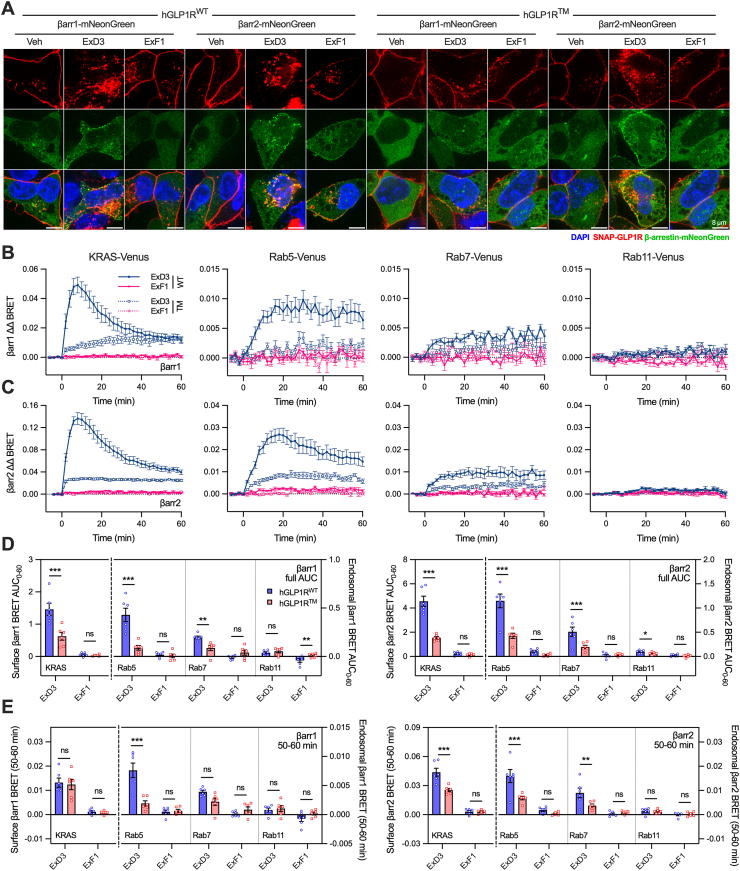
**Endosomal β-arrestin recruitment is influenced by GLP-1R C-tail phosphomutation.** (**A**) Representative images (*n* = 3) showing stable SNAP-hGLP1R^WT^ or SNAP-hGLP1R^TM^ cells transduced with β-arrestin-1- or -2-mNeonGreen, 30 min after stimulation with vehicle or 1 μM agonist, scale bar = 8 μm. (**B**) 1 μM agonist-induced β-arrestin-1-Nluc recruitment to plasma membrane (KRAS-Venus), early endosomes (Rab5-Venus), late endosomes (Rab7-Venus) or recycling endosomes (Rab11-Venus) over 60 min in SNAP-hGLP1R^WT^ or SNAP-hGLP1R^TM^ cells, *n* = 6. (**C**) As for (B) but β-arrestin-2-Nluc. (**D**) Quantification of full 60-minute stimulation AUCs from (B) and (C) with comparisons by two-way matched ANOVA with Šidák's test. Note that KRAS is plotted using the left-hand y-axes and the Rabs are plotted using the right-hand y-axes. (**E**) Quantification of steady state β-arrestin-1 or -2 recruitment, measured as the mean signal from the final 10 min of incubation from (B) and (C), with comparisons by two-way matched ANOVA with Šidák's test. KRAS is plotted using the left-hand y-axes and the Rabs using the right-hand y-axes. ∗p < 0.05, ∗∗p < 0.01, ∗∗∗p < 0.001 by indicated statistical test. All data shown as mean ± SEM, with individual replicates in some cases.

Given the impact of the triple mutation on GLP-1R internalisation and post-endocytic trafficking, we aimed to investigate how this affects the subcellular localisation and kinetics of β-arrestin coupling. GLP-1R has been described as showing class A behaviour with regard to β-arrestin recruitment, i.e. β-arrestin dissociates as the receptor internalises [44]. Indeed, after treatment with ExD3 or GLP-1, β-arrestin-1 and -2 association to the hGLP-1R and mGLP-1R peaked at 5 min and then declined, consistent with the kinetics of internalisation (Supplementary Fig. 3A–B). However, we also observed that dynamic punctate aggregates of β-arrestin-2-mNeonGreen were found not only at the plasma membrane but in the intracellular space 30 min after treatment with GLP-1 in stable SNAP-hGLP1R^WT^ cells (Supplementary Video 2). Static images of cells fixed at this time-point suggested extensive endosomal β-arrestin-2 localisation with ExD3, but not ExF1 (Fig. 3A). Some β-arrestin recruitment was apparent with ExD3 in SNAP-hGLP1R^TM^ cells under the same conditions but appeared more localised to the plasma membrane. β-arrestin-1 followed a similar pattern, but the amount of recruitment appeared to be less compared to with β-arrestin-2 (Fig. 3A).

Supplementary video related to this article can be found at https://doi.org/10.1016/j.molmet.2026.102321

The following is/are the supplementary data related to this article:

Supplementary Video 2 Supplementary Video 2

To quantify β-arrestin recruitment at different subcellular locations we performed bystander BRET assays using cell surface/endosome markers. These experiments confirmed the rise-and-fall pattern of both β-arrestin isoforms at the plasma membrane with ExD3 in SNAP-hGLP1R^WT^ cells, and notably also showed that ExD3 can also drive or maintain recruitment at early (Rab5) and late (Rab7) endosomal compartments (Fig. 3B, C, Supplementary Fig 3C–E). There were very small but still detectable responses at recycling (Rab11) endosomes for β-arrestin-2 but not β-arrestin-1. Compared to at the plasma membrane, endosomal responses were more persistent over time. All ExD3 responses were clearly reduced with hGLP1R^TM^ compared to hGLP1R^WT^, with AUC calculation from the full 60-minute stimulation period indicating that the overall loss of recruitment at hGLP1R^TM^ was proportionate at each location, i.e. approximately 60–70% less than that seen with hGLP1R^WT^ (Fig. 3D). However, later in the incubation period (i.e. corresponding more closely to steady state pharmacological stimulation), β-arrestin responses with hGLP1R^TM^ were in fact relatively preserved at the cell surface compared to endosomes (Fig. 3E), presumably due to greater retention of the activated GLP-1R at this location. For example, the hGLP1R^TM^ β-arrestin-1 responses to ExD3 during the final 10 minutes of the stimulation were reduced by only 10% at the plasma membrane but by 75% at early endosomes, compared to with hGLP1R^WT^.

These findings show that 1) β-arrestins continue to associate with the GLP-1R after internalisation, and 2) hGLP1R^TM^ leads to a relative shift in the localisation of β-arrestin recruitment towards the plasma membrane and away from endosomes, likely due to slower internalisation.

### 2.4 Loss of three GLP-1R phosphorylation sites alters the subcellular localisation of Gα_s_ activation

**Figure 4:**
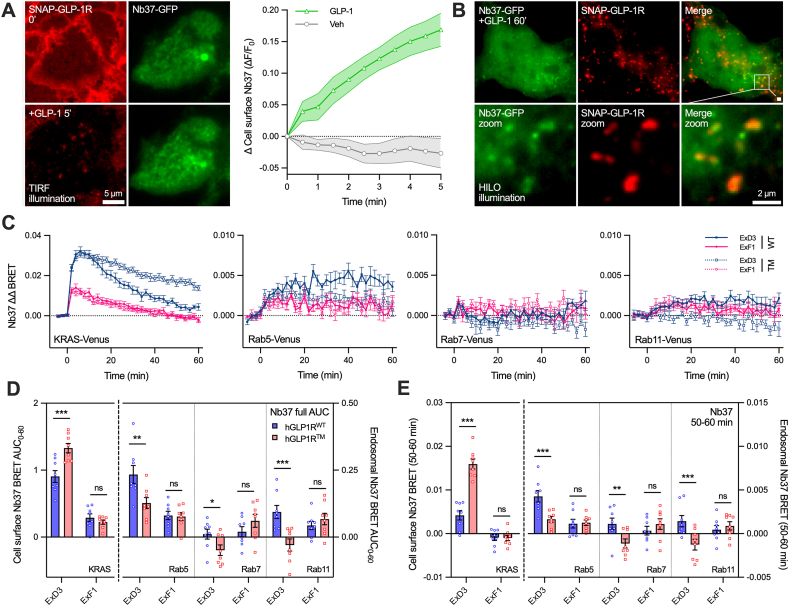
**The spatial organisation of Gα_s_ activation is influenced by GLP-1R C-tail phosphomutation.** (**A**) TIRF images and quantification of 1 μM GLP-1-induced Nb37-GFP plasma membrane recruitment time-course in SNAP-hGLP1R^WT^ cells, *n* = 5. (**B**) Representative image showing colocalisation of Nb37-GFP puncta with internalised SNAP-GLP-1R 60 min after treatment with 1 μM GLP-1. (**C**) 1 μM agonist-induced Nb37-Nluc recruitment time-course to indicated location in SNAP-hGLP1R^WT^ or SNAP-hGLP1R^TM^ cells, *n* = 8. (**D**) Quantification of full 60-min stimulation AUC from (C) with comparisons by two-way matched ANOVA with Šidák's test. (**E**) Quantification of steady state Nb37 response, measured as the mean signal from the final 10 min of incubation from (C), with comparisons by two-way matched ANOVA with Šidák's test. ∗p < 0.05, ∗∗p < 0.01, ∗∗∗p < 0.001 by indicated statistical test. All data shown as mean ± SEM, with individual replicates in some cases.

Given the changes to β-arrestin localisation seen with hGLP1R^TM^, we also investigated its effects on endogenous Gα_s_ signalling using nanobody-37 (Nb37), which detects the open, active form of Gα_s_ [45]. Using TIRF microscopy we first verified that GLP-1R activation by GLP-1 leads to an increase in localisation of transfected GFP-tagged Nb37 to the plasma membrane in stable hGLP1R^WT^ cells (Fig. 4A, Supplementary Fig. 4A). Moreover, with longer stimulations we observed the presence of Nb37-GFP associated with internalised SNAP-GLP-1R-containing endosomes (Fig. 4B).

To quantify these processes we used bystander BRET to detect agonist-mediated translocation of nanoluciferase-tagged Nb37 to the plasma membrane and Rab5/7/11 endosomes. Here, both ligands produced initial peaks of Gα_s_ activation at the plasma membrane, which were similar in both stable SNAP-hGLP1R^WT^ and SNAP-hGLP1R^TM^ cells, followed by a decline (Fig. 4C). As a partial agonist, ExF1 produced lower peak Gα_s_ activation than ExD3. For ExD3 the decline with hGLP1R^TM^ was slower than with hGLP1R^WT^, in keeping with slower internalisation of the phosphodeficient triple mutant receptor and leading to a greater cumulative plasma membrane Gα_s_ response when quantified as AUC from the full 60-minute stimulation (Fig. 4D); this difference was accentuated by considering the response at steady state i.e. the final 10 minutes (Fig. 4E). The ExD3/hGLP1R^TM^ combination showed a similar rate of decline as ExF1/hGLP1R^WT^ (Supplementary Fig. 4B).

Gα_s_ activation was also detected at Rab5-positive early endosomes and, contrasting with the cell surface response, was larger with hGLP1R^WT^ than hGLP1R^TM^ for ExD3. Early endosomal Gα_s_ activation was also detectable with ExF1 in hGLP1R^WT^ cells, with a similarly reduced magnitude to that at the plasma membrane (i.e. a ∼70% reduction compared to with ExD3). This is noteworthy as it contrasts with an expectation that the relative lack of GLP-1R internalisation with ExF1 would further suppress its Gα_s_ signalling efficacy at this location. ExF1-induced endosomal Gα_s_ signalling was not affected by the triple mutation. There was little evidence of agonist-induced Gα_s_ activation in late endosomes; in fact there was with a small reduction in BRET signal seen with ExD3 compared to vehicle, particularly with hGLP1R^TM^, which we speculate results from Nb37 redistribution away from the Rab7 compartment to other sites of GLP-1R activation. Recycling endosome responses were small but detectable, and again showed a slight negative BRET signal with ExD3 at hGLP1R^TM^.

These data show how the location of Gα_s_ activation can be shifted towards the plasma membrane by loss of GLP-1R C-terminal phosphorylation.

### 2.5 Loss of three GLP-1R phosphorylation sites increases cAMP in a ligand-specific manner and selectively increases kinase signalling

**Figure 5:**
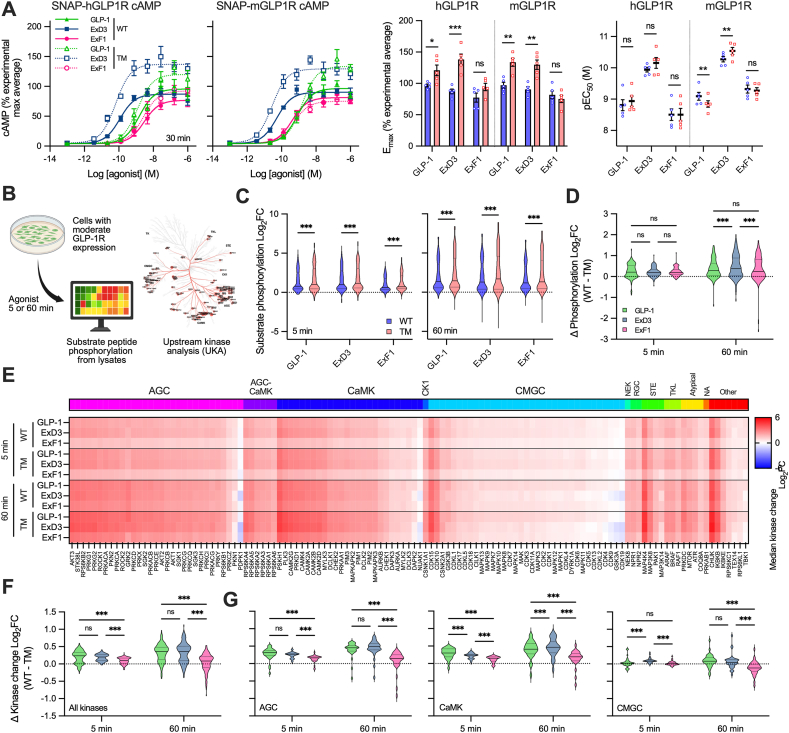
**Signalling responses are enhanced with the triple phosphomutant GLP-1R.** (**A**) cAMP accumulation in AD293 cells expressing SNAP-GLP1R and stimulated for 30 min with agonist, *n* = 5. E_max_ and pEC_50_ values compared by two-way matched ANOVA with Šidák's test. (**B**) Schematic representing the workflow for the kinome assay. (**C**) Violin plots showing the global effect of 1 μM agonist compared to vehicle on substrate phosphorylation in the kinome assay, *n* = 5. (**D**) The effect of the triple mutation on phosphorylation responses, determined by subtracting the hGLP1R^WT^ log_2_ fold change for each substrate from that of hGLP1R^TM^, with comparison by two-way matched ANOVA with Tukey's test. (**E**) Heatmap showing the results of the upstream kinase analysis (UKA), with predicted kinase activities shown as the median log_2_ kinase change. (**F**) The effect of the triple mutation on overall predicted kinase activity, determined by subtracting the hGLP1R^WT^ log_2_ fold change for each kinase change from that of hGLP1R^TM^, with statistical comparison by two-way matched ANOVA with Tukey's test. (**G**) As for (F) but considering AGC, CaMK and CMGC groups separately. ∗p < 0.05, ∗∗p < 0.01, ∗∗∗p < 0.001, by indicated statistical test. All data shown as mean ± SEM, with individual replicates in some cases.

There is debate about whether β-arrestins and GPCR endocytosis are net negative or net positive regulators of intracellular signalling, particular in view of the role of internalised agonist/receptor complexes as signalling hubs. We aimed to use the phosphorylation deficient receptor model to address this question for GLP-1R, as data thus far show it reduces β-arrestin recruitment and GLP-1R internalisation, and switches the location of Gα_s_ signalling towards the plasma membrane and away from endosomes. We found that GLP1R^TM^ led to increased maximal whole cell cAMP accumulation after 30-minute stimulation with GLP-1 or ExD3, compared to GLP1R^WT^, but not with ExF1 (Fig. 5A). These data suggest β-arrestin recruitment, GLP-1R internalisation, or both, limit overall GLP-1RA-mediated cAMP generation, and provide support for the notion that GLP-1R at the cell surface is a more efficient source of cAMP generation than internalised GLP-1R [46].

β-arrestins can act as scaffolds to initiate activation of kinase networks, including for GLP-1R [9,10], raising the question of whether loss of β-arrestin interactions would lead to more selective kinomic responses with the phosphorylation mutant receptor. To investigate we used the PamChip kinome array to measure activation of a range of serine–threonine kinases (STKs) in AD293 cells transfected with hGLP1R^WT^ or hGLP1R^TM^. This system measures the ability of agonist-treated cell lysates to phosphorylate a panel of kinase substrate peptides, allowing upstream kinase activity to be inferred. Previous studies have used this approach to study GLP-1R signalling in EndoC-βH1 and INS-1 832/3 cells [38,46]. In the present work we compared 5- and 60-minute stimulations to probe acute and sustained responses, respectively, and a high agonist concentration (1 μM) to avoid affinity-related differences (Fig. 5B). Robust agonist responses were observed, as shown as individual substrate phosphorylation signals in Supplementary Fig. 5A. When the log_2_ fold change *versus* vehicle for each phosphosite were considered as a whole, hGLP1R^TM^ led to larger responses with all three agonists at both timepoints (Fig. 5C), but it was notable that the difference was largest for ExD3 at 60 minutes (Fig. 5D), in keeping with the cAMP data in Fig. 5A.

Upstream kinase analysis (UKA) was performed to predict activity of kinases that could have produced the observed pattern on substrate phosphorylation by functional class scoring. The results of this analysis are shown as a heatmap in Fig. 5E with predicted activities of 101 kinases sorted into groups. GLP-1 and ExD3 showed very similar patterns of predicted activity at both hGLP1R^WT^ and hGLP1R^TM^, with ExF1 showing globally reduced responses at the acute timepoint, in line with its partial agonist characteristics. However, after 60 minutes, ExF1 responses were close to the other two agonists. By subtracting the predicted kinase changes at hGLP1R^WT^ and hGLP1R^TM^ at each time-point, it was apparent that the triple mutant resulted in generally higher kinase activity for all ligands at both time-points, although the effects were larger for GLP-1 and ExD3 than ExF1, and were also larger at the 60-minute time-point than at 5 minutes (Fig. 5F). The magnitude of the average difference between hGLP1R^WT^ and hGLP1R^TM^ responses was similar to the ∼50% increase in cAMP accumulation seen with hGLP1R^TM^/ExD3 in Fig. 5A. However, when the three largest kinase groups (AGC, CaMK, CMGC) were considered separately, it was noted that the effect of GLP1R^TM^ to increase CMGC responses was reduced compared to the other two groups. This might be explained by loss of β-arrestin-mediated kinase activation, which has been proposed for CMGC group kinases such as ERK1/2 [10].

### 2.6 Validation of triple phosphomutation effects in pancreatic islets

**Figure 6:**
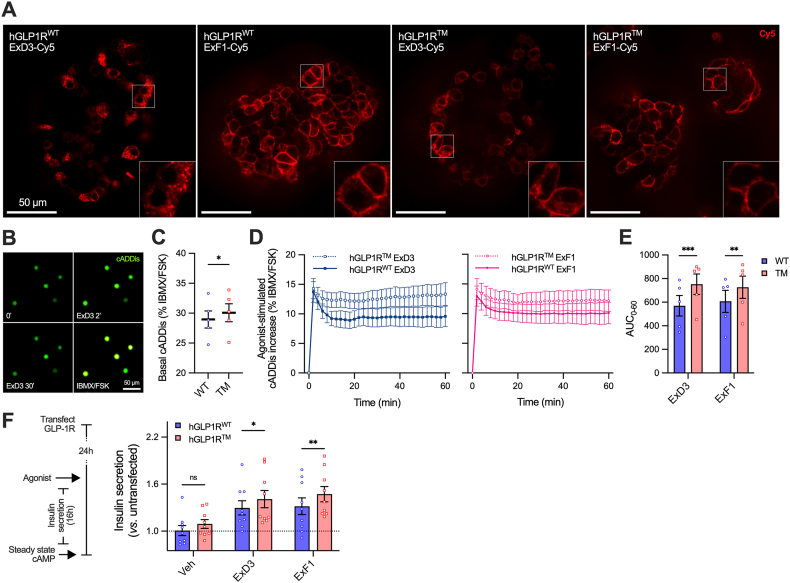
**GLP-1R triple phosphomutant responses in a pancreatic islet context.** (**A**) Confocal images of *Glp1r* KO islets transduced for 16 h with hGLP1R^WT^ or hGLP1R^TM^ adenoviral particles and treated with 100 nM ExD3-Cy5 or ExF1-Cy5 for 30 min before imaging. Representative of *n* = 3 mice. Scale bar = 50 μm. See also Supplementary Fig. 6A. (**B**) Representative images showing cADDis signal in *Glp1r* knockout islet cells transduced with hGLP1R^WT^. (**C**) Basal cADDis signal from (B), *n* = 5, comparison by paired t-test. (**D**) Dynamic cADDis cAMP responses to 100 nM agonist, *n* = 5. (**E**) AUC analysis of (D) with comparison by two-way matched ANOVA with Šidák's test. (**D**) Schematic and results of sustained (16 h) insulin secretion in response to 100 nM agonist using hGLP1R-transfected INS-1 cells with knockout of endogenous GLP-1R, *n* = 9, comparison by two-way matched ANOVA with Šidák's test. ∗p < 0.05, ∗∗p < 0.01, ∗∗∗p < 0.001 by indicated statistical test. All data shown as mean ± SEM, with individual replicates in some cases.

The effects of pathway selectivity can be tissue-specific due to differences in expression of signalling transducers and regulators. Pancreatic β-cells have highly adapted cAMP signalling machinery to ensure tight coupling of changes in glycaemic status to insulin release [47]. To examine the effect of the phosphorylation deficient GLP-1R mutant in a pancreatic islet context we used adenoviral expression of untagged hGLP1R^WT^ and hGLP1R^TM^ in islets from *Glp1r* knockout mice in conjunction with fluorescent Cy5 conjugates of ExF1 and ExD3 [46]. The expected pattern of ligand internalisation was observed, with ExD3-Cy5 showing extensive uptake with hGLP1R^WT^ but partial membrane localisation with hGLP1R^TM^; ExF1-Cy5 showed little internalisation at both receptor forms (Fig. 6A, Supplementary Fig. 6A).

To assess signalling responses at each receptor in an islet context we combined adenoviral GLP-1R expression with the Epac-based cADDis cAMP sensor [48] in dispersed islet cells from *Glp1r* knockout mice. In this experiment, high throughput imaging allows cAMP dynamics to be recorded from many hundreds of cells for each condition in parallel (Fig. 6B). Using the sensor saturation response to forskolin as a reference, hGLP1R^TM^ showed a modestly increased basal cAMP level compared to hGLP1R^WT^ (Fig. 6C). When stimulated by 100 nM ExD3 and ExF1, both wild-type and mutant receptors showed similar peak responses, but there was a greater subsequent decline in cAMP levels over time with hGLP1R^WT^, particularly in response to ExD3 (Fig. 6D, Supplementary Fig. 6B and 6C). This translated to greater cumulative cAMP exposure with the mutant (Fig. 6E).

We also evaluated the effect of the phosphosite triple mutation on coupling to insulin secretion by transfecting hGLP1R^WT^ or hGLP1R^TM^ into INS-1 832/3 cells that lack endogenous GLP-1R expression after deletion by CRISPR/Cas9 [49]. In these assays cells were stimulated for 16 hours with agonist, as this mimics pharmacological agonist exposure more closely than acute stimulation and can help to unveil the impact of GLP-1R desensitisation [13]. Here hGLP1R^TM^ resulted in higher insulin release than hGLP1R^WT^ (Fig. 6F). However, this effect was not agonist-specific. We also note that ExF1 did not produce a greater insulin secretory response than ExD3 in these experiments, contrasting with results obtained previously in isolated β-cells expressing GLP-1R endogenously and *in vivo* [13,15]; we speculate this could reflect the impact of increased GLP-1R density seen with overexpression in the current study.

### 2.7 Relative impacts of GLP-1R β-arrestin recruitment and internalisation on cAMP signalling

**Figure 7:**
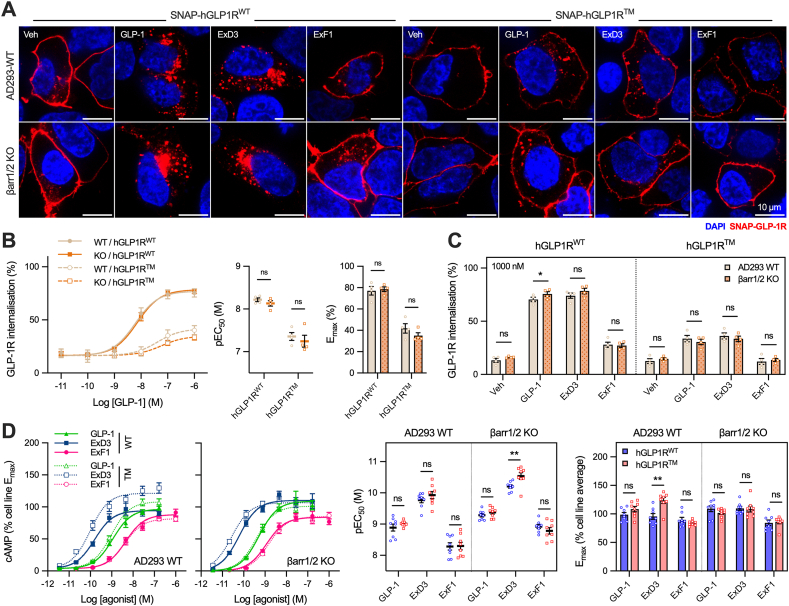
**GLP-1R triple phosphomutant enhances cAMP signalling in the absence of β-arrestins.** (**A**) Representative images showing wild-type or β-arrestin-1/2 knockout AD293 cells expressing SNAP-hGLP1R^WT^ or SNAP-hGLP1R^TM^ 30 min after stimulation with vehicle or 1 μM agonist, scale bar = 10 μm. (**B**) GLP-1R internalisation at 30 min in wild-type or β-arrestin-1/2 knockout AD293 cells measured via reversible SNAP-GLP1R labelling, *n* = 4, E_max_ and pEC_50_ values compared by two-way matched ANOVA with Šidák's test. (**C**) GLP-1R internalisation in response to 1 μM agonist at 30 min in wild-type or βarr1/2 knockout AD293 cells measured via reversible SNAP-GLP1R labelling, *n* = 4, with comparison by two-way matched ANOVA with Šidák's test. (**D**) cAMP accumulation wild-type or β-arrestin-1/2 knockout AD293 cells expressing SNAP-hGLP1R^WT^ or SNAP-hGLP1R^TM^ and stimulated for 30 min with agonist, *n* = 8. E_max_ and pEC_50_ values compared by two-way matched ANOVA with Šidák's test. ∗p < 0.05, ∗∗p < 0.01 by indicated statistical test. Data shown as mean ± SEM with individual replicates.

Data from GLP1R^TM^ experiments thus far support a paradigm in which internalisation and/or β-arrestin recruitment serve mainly as negative regulators of GLP-1R downstream signalling, but the contribution of each process cannot be separately ascertained. We therefore aimed to distinguish the relative impact of each by first eliminating the contribution of β-arrestins using dual β-arrestin-1/2 knockout cells [50]. In keeping with earlier data showing that β-arrestins are not required for GLP-1R endocytosis [11,13,[23], [24], [25],^51^], internalisation responses with GLP-1, ExD3 or ExF1 were unchanged by the absence of β-arrestins with both hGLP1R^WT^ and hGLP1R^TM^ (Figure 7A–C).

Similar to results shown in Fig. 5, measurements of cAMP accumulation in wild-type AD293 cells showed that ExD3 (but not ExF1) produced an increase in maximal response at hGLP1R^TM^ compared to hGLP1R^WT^ (Fig. 7D). Matched experiments in β-arrestin-1/2 knockout cells showed an increase in potency with ExD3 at hGLP1R^TM^, although not in maximal response. This signalling advantage of hGLP1R^TM^ was not due to higher receptor density, which was actually slightly lower than for hGLP1R^WT^ in both cell lines in this set of experiments (Supplementary Fig. 7A).

These results appear to suggest that the effect of the phosphorylation mutations to increase cAMP signalling with ExD3 is not via loss of β-arrestin recruitment.

### 2.8 Differentiated roles of three putative GLP-1R phosphorylation sites on β-arrestin recruitment and internalisation

**Figure 8:**
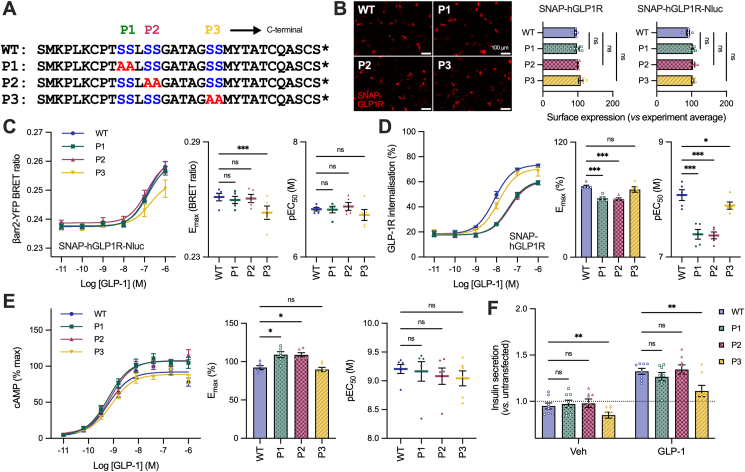
**Selective effects of individual phosphosites on GLP-1R internalisation and β-arrestin recruitment.** (**A**) P1, P2 and P3 C-tail mutation sequences. (**B**) Surface expression using SNAP-tag labelling in AD293 cells, *n* = 5, with one-way matched ANOVA and Dunnett's test comparing mutation effects. (**C**) GLP-1-induced β-arrestin-2-YFP recruitment at 5 min, *n* = 6, two-way matched ANOVA with Dunnett's test for E_max_ and pEC_50_ comparisons. (**D**) GLP-1-induced GLP-1R internalisation at 30 min in AD293 cells measured via reversible SNAP-GLP1R labelling, *n* = 5, E_max_ and pEC_50_ compared by two-way matched ANOVA with Dunnett's test. (**E**) cAMP accumulation in AD293 cells expressing SNAP-hGLP1R and stimulated for 30 min with agonist, *n* = 6, two-way matched ANOVA with Dunnett's test for E_max_ and pEC_50_ comparisons. (**F**) Sustained (16 h) insulin secretion results in response to 100 nM GLP-1 using hGLP1R-transfected INS-1 cells with knockout of endogenous GLP-1R, *n* = 8, comparison by two-way matched ANOVA with Šidák's test. ∗p < 0.05, ∗∗p < 0.01, ∗∗∗p < 0.001 by indicated statistical test. All data shown as mean ± SEM, with individual replicates in some cases.

To further probe the relative impacts of β-arrestin recruitment and GLP-1R internalisation we generated alanine mutants of the three individual phosphosites in the human SNAP-GLP1R (hGLP1R^P1^, hGLP1R^P2^, hGLP1R^P3^; Fig. 8A). All these variants, with and without nanoluciferase C-terminal tags, were expressed at wild-type-like levels at the plasma membrane when transiently expressed in AD293 cells (Fig. 8B).

GLP-1-induced β-arrestin-2 responses were reduced with the distal hGLP1R^P3^ mutant, as detected by BRET-based recruitment measurements and Borealis activation sensor (Fig. 8C, Supplementary Fig. 8A and 8B). In contrast, and in spite of the two proximal phosphorylation sites conforming to the “PXPP” motif linked to β-arrestin engagement [52], the hGLP1R^P1^ and hGLP1R^P2^ mutants showed only minor loss of β-arrestin responses, although hGLP1R^P2^ still produced a statistically significant reduction in the activation assay. Interestingly, the opposite trend was seen for GLP-1R internalisation, which was clearly slower and showed reduced potency with hGLP1R^P1^ and hGLP1R^P2^ over a 30-minute stimulation period, whereas hGLP1R^P3^ showed the smallest difference compared to the wild-type (Fig. 8D, Supplementary Fig. 8C).

As the triple hGLP1R mutant had showed increased cAMP accumulation (see Fig. 5A) we wondered about the signalling impact of individual phosphosite mutations. Although the differences were not as large, there were statistically significant increases in maximal GLP-1-induced cAMP accumulation with the two mutants with reduced GLP-1R internalisation tendency, i.e. hGLP1R^P1^ and hGLP1R^P2^, but not with hGLP1R^P3^ (Fig. 8E). However, when transiently expressed in INS-1 cells without endogenous GLP-1R expression, neither hGLP1R^P1^ nor hGLP1R^P2^ resulted in increases in insulin secretion in response to sustained exposure to GLP-1 (Fig. 8F). Paradoxically hGLP1R^P3^ led to reduced insulin secretion, both with and without GLP-1.

Given that the GLP-1R C-tail and helix 8 contain several other serine or threonine residues that can potentially act as phosphorylation acceptors, we generated constructs with all additional serine/threonines mutated to alanine, both with (hGLP1R^all^) and without (hGLP1R^extra^) the P1/2/3 alanine mutations (Supplementary Fig. 8D). Unlike hGLP1R^TM^, both hGLP1R^all^ and hGLP1R^extra^ showed poor surface expression (Supplementary Fig. 8E). GLP-1-induced percentage GLP-1R internalisation was not substantially altered with hGLP1R^extra^; surface expression of hGLP1R^all^ was too low to reliably detect changes in this assay (Supplementary Fig. 8F). cAMP responses for hGLP1R^extra^ and hGLP1R^all^ were reduced in line with lower receptor density (Supplementary Fig. 8G).

Overall, these data point to differentiated roles of specific sites in the GLP-1R C-terminus to drive receptor internalisation *versus* β-arrestin responses, with additional evidence suggesting that slowing of internalisation, rather than reducing β-arrestin recruitment, may lead to enhanced signalling potential, in agreement with results in Figure 7.

## 3 Discussion

In this study we aimed to establish the cellular consequences of disrupting three previously identified serine phosphorylation sites in the GLP-1R C-tail. This question has taken on renewed importance given the interest in using G protein-biased agonism to increase therapeutic targeting of this GPCR in obesity and T2D. Distinct from earlier studies [[31], [32], [33]] we have integrated ligands with different signalling profiles to help triangulate the impact of the selected C-tail mutations and assess the suitability of this approach to model G protein-biased agonism. Our key findings are that 1) combined loss of all three phosphorylation sites inhibits β-arrestin recruitment and slows GLP-1R internalisation, driving preferential Gα_s_ signalling at the plasma membrane rather than endosomes; 2) cAMP accumulation and other downstream signalling responses are enhanced when β-arrestin recruitment and GLP-1R internalisation are reduced in this manner; 3) these effects are accentuated by ligands that ordinarily show higher levels of β-arrestin recruitment and GLP-1R internalisation (i.e. GLP-1 and ExD3); 4) slowing internalisation appears more important than reducing β-arrestin recruitment for the signalling-enhancing effect of the phosphodeficient GLP-1R; and 5) similar effects are seen in both human and mouse GLP-1R models. Overall, these data support the potential for G protein bias to improve GLP-1R signalling efficacy.

An early study used incorporation of radiolabelled orthophosphate to identify phosphorylatable residues in the GLP-1R C-tail [31], with recent data validating the same sites using immunolabelling [53]. These approaches are indirect as they rely on demonstrating that total phosphorylation is reduced when the candidate phosphosites are removed or mutated. In recent years direct measurement of GPCR phosphorylation by mass spectrometry has become possible, e.g. for the GIP and ghrelin receptors [54,55]. However, until recently this had not been accomplished for the GLP-1R. In 2024 Lamb et al. [33] described a mass spectrometry approach to study GLP-1R C-tail phosphorylation, identifying S442, S444, and S445 as the specific serines that undergo agonist-mediated phosphorylation. These correspond to the proximal P1 and P2 sites from our study. Of note, compared to GIPR and glucagon receptor (GCGR), measurement of GLP-1R phosphorylation was a greater technical challenge, partly due to a relatively low abundance of phosphorylated GLP-1R despite agonist stimulation. A modified GLP-1R construct was generated to facilitate detection, in which a proteolytic cleavage site was inserted between the distal end of helix 8 and the C-tail to allow an extra enrichment step before analysis. The functional effects of alanine mutation of the identified serines were also tested, with only minor effects on β-arrestin recruitment and GLP-1R internalisation seen with individual mutations, and a larger but still moderate inhibitory effect when all three were mutated in combination; specifically, the S442/444/445A mutation produced an approximate 50% reduction in maximal β-arrestin-2 recruitment and a three-fold reduction in GLP-1R internalisation potency. Although the mutations used in our study are not identical, we found that the proximal P1 and P2 sites had only an equivocal role in β-arrestin recruitment but had a larger effect on GLP-1R internalisation. Differences in receptor constructs and assay design may have contributed to these inconsistencies. We note that a larger role in GLP-1R internalisation compared to β-arrestin recruitment for the proximal P1/P2 region has been hinted at in other studies [31,56]. The fact that agonist-induced GLP-1R phosphorylation at the more distal (P3) GLP-1R site was not detected by mass spectrometry [33] is notable, as alanine mutation at this position inhibits β-arrestin recruitment [56], including in our study. Given the challenges of measuring GLP-1R phosphorylation, it is possible that phosphorylation at these residues was in fact present, but below the limit of detection. Some support for this possibility is offered by the observation that phosphorylation of additional (but unidentified) GLP-1R C-tail serine residues can be provoked when the canonical S442/444/445 residues are disrupted [33]. Another possibility is that these processes could be cell-specific due to altered expression patterns of GRK and other regulatory proteins.

Our results comparing ExF1 at the wild-type GLP-1R with GLP-1 or ExD3 at the triple mutant GLP-1R make possible direct comparisons of ligand- and receptor-mediated biased agonism. β-arrestin recruitment, GLP-1R internalisation and post-endocytic trafficking properties of these approaches generally converged well. The main difference was in the magnitude of the G protein activation and cAMP responses, with ExF1 as expected behaving as a partial agonist but the triple mutant receptor retaining full G protein activation, in keeping with the fact that the GLP-1R C-tail is not directly involved in G protein engagement. This difference is potentially an advantage as most biased GLP-1RAs show partial G protein agonism [3,15,16,39], meaning researchers in the field lack tools to study “pure” G protein-biased agonism. Our results suggest development of improved biased GLP-1RAs that retain full G protein efficacy may enhance therapeutic efficacy by increasing signalling.

G protein-biased GLP-1RAs typically show reduced β-arrestin recruitment and GLP-1R internalisation, but many studies have shown that these events proceed independently of each other [13,[21], [22], [23], [24]]. This invites the question of whether the apparent benefits of G protein-biased GLP-1R agonism depend more on inhibiting one of these processes than the other. Here, we have found that the ability of the phosphodeficient GLP1R^TM^mutant to enhance cAMP accumulation is preserved in β-arrestin-1/2 knockout cells. We also found that the GLP-1R internalisation-reducing GLP1R^P1^ and GLP1R^P2^ mutants increased GLP-1-induced cAMP accumulation, but the β-arrestin recruitment-reducing GLP1R^P3^ did not. Together, these observations point to a larger role for GLP-1R internalisation in negatively regulating agonist-mediated cAMP accumulation. This is perhaps surprising, as a role for internalised GLP-1R signalling to perpetuate cAMP generation is well established in the field [12,57,58]. However, we have found that the contribution of internalised GLP-1R signalling to overall cAMP accumulation is fairly modest under sustained stimulation with pharmacological agonists [46]. On the other hand, slower overall internalisation might correspond to a relative increase in the time that the receptor is resident in signalling-competent endosomes compared to the usual rapid transit to lysosomal degradation [13]. It is also important to note that changes in overall cellular cAMP might not correspond to potential mutation-specific effects on GLP-1R signalling within highly localised nanodomains, which are thought to be operational in β-cells [59] and are likely to be influenced by the large differences in GLP-1R redistribution consequent to loss of C-tail phosphorylation.

Our finding that loss of C-tail phosphorylation has similar functional effects at both human and mouse GLP-1R provides indirect evidence that GLP-1R biased agonism is likely to be a translatable phenomenon. Any such data is potentially valuable, as no study directly comparing oppositely biased but otherwise matched GLP-1RAs in humans has been reported to date. Whilst there are increasing numbers of G protein-biased GLP-1RAs approved for human use, or in late-stage clinical development, differences in dosing and ligand pharmacokinetics make it difficult to unambiguously link clinical profiles to biased signalling. Therefore, most experimental support for this approach derives from rodent studies. Many GPCRs show species divergence, with a relevant example being the differences in phosphorylation sites found in human and mouse GIPR C-tails, which has a significant bearing on the suitability of mice for studying GIPR biased agonism [60]. Nevertheless, there is still a pressing need for carefully designed experimental medicine studies to resolve the question of whether G protein-biased GLP-1R agonism is a valid approach for humans [61].

Some experimental limitations in our study include the fact that we did not verify the effect of each mutation on phosphorylation directly, we only used alanine mutations and did not test phosphomimetic mutations e.g. aspartate or lysine, we only assessed known phosphorylation sites in the C-tail, and potentially relevant phenomena such as ligand binding, receptor anchoring or trafficking to nanodomains were not included in our pharmacological evaluation. Moreover, all experiments used exogenous GLP-1R expression and much of the work was performed in HEK293 cells, we did not examine how these processes might differ in diverse GLP-1R-expressing cell types, and we focussed on pharmacological stimulations which might not recapitulate how the receptor behaves in response to its endogenous ligands. Indeed, given the short half-lives and picomolar circulatory concentrations of native GLP-1 (7–36)NH_2_, it is quite possible that β-arrestin recruitment and GLP-1R internalisation initially enhance signalling from internal compartments; in conjunction with the need to exert fine temporal control over receptor signalling, this may well be why evolution has preserved a GLP-1R sequence that exhibits these behaviours. These topics could be investigated in future studies, with development of cellular or animal models with mutations of interest introduced into the endogenous *GLP1R* locus a logical next step to allow more physiological and therapeutic evaluations. Nevertheless, our results provide a body of evidence that receptor-mediated slowing of GLP-1R internalisation can enhance net GLP-1R signalling in a pharmacological setting, thereby providing conceptual support for the use of G protein-biased agonism to increase GLP-1R agonist efficacy.

## 4 Methods

### 4.1 Peptides

GLP-1 (7–36)NH_2_, ExD3 and ExF1 were purchased WuXi AppTec, China. ExD3-Cy5 and ExF1-Cy5 were generated previously [46].

### 4.2 Plasmid and viral vectors

pcDNA5/FRT/TO (Thermo Fisher, UK) encoding human or mouse GLP-1R (wild-type or with the described mutations) with an N-terminal SNAP-tag and custom signal peptide, and the same but featuring C-terminal nanoluciferase, were generated by Genewiz, UK; these plasmids were used for all transient GLP-1R transfections. In the nanoluciferase-tagged constructs the luciferase was fused in frame with the GLP-1R C-terminus without an intervening linker. The same inserts (without the nanoluciferase) were also generated in the pcDNA5/FRT vector, and used for stable cell line generation. β-arrestin-1-Nluc, β-arrestin-2-Nluc and Nb37-Nluc were also generated by Genewiz in the pcDNA5/FRT vector. KRAS-Venus, Rab5-Venus, Rab7-Venus and Rab11-Venus were kindly provided by Nevin Lambert (Augusta University, USA) and Kevin Pfleger (University of Western Australia). Nb37-GFP was a gift from Roshanak Irannejad (UCSF, USA). BacMam-encoded cADDis cAMP sensor (U0250G), Borealis β-arrestin-2 sensor (D2020G) and β-arrestin-1- and -2-mNeonGreen (custom orders) were obtained from Montana Molecular, USA. Custom adenoviruses encoding non-tagged hGLP1R^WT^ and hGLP1R™ plus a tdTomato transduction reporter, or control adenovirus encoding EGFP, were obtained from VectorBuilder, UK.

### 4.3 Cell lines and cell culture

Adherent HEK293 cells (AD293) were cultured in DMEM supplemented with 10% foetal bovine serum (FBS) and 1% penicillin/streptomycin. Dual β-arrestin-1/2 knockout cells was described previously [50]. Stable Flp-In cells expressing SNAP-hGLP1R^WT^ or SNAP-hGLP1R^TM^ were generated by co-transfection of pcDNA5/FRT-SNAP-GLP-1R plasmids with pOG44 in a 1:10 ratio into Flp-In 293 cells and selection using hygromycin B. These cells do not require tetracycline to induce gene expression. INS-1 832/3 cells with deletion of the endogenous GLP-1R by CRISPR/Cas9 [49], a gift from Jacqueline Naylor (AstraZeneca, UK), and were cultured in RPMI-1640 with 10% FBS and 1% penicillin/streptomycin at 11 mM glucose.

### 4.4 Animals

Experiments were approved by the Animal Welfare and Ethical Review Board at Imperial College London and conducted under the UK Animal (Scientific Procedures) Act 1986 with a Home Office Project Licence. Whole body *Glp1r* knockout mice were generated by crossing β-actin-Cre mice [62] (JAX strain #:033984) with *Glp1r*^fl/fl^ mice [63] (JAX strain #:035238), kindly provided by Prof Randy Seeley (University of Michigan) to delete *Glp1r* in the germline. Mice were kept on a 12-hour light/dark cycle with ad libitum access to standard chow (RM1(E), Special Diets Services, UK) and water.

### 4.5 Measurement of SNAP-GLP-1R surface density

Stable SNAP-hGLP1R-expressing Flp-In cells, or AD293 cells reverse transfected with SNAP-GLP1R for 24 h using Lipofectamine 2000, were seeded in black-walled, clear-bottomed plates (Greiner, UK) coated with 0.01% poly-d-lysine. Cells were labelled with SNAP-Surface AlexaFluor-647 (500 nM, obtained from New England Biolabs, UK) for 10 min at 37 °C, and, after washing three times with PBS, imaged live using an automated Nikon Ti2-E-based microscope (Cairn Research, UK) equipped with a LED source (CoolLED, UK), pco.edge 4.2 bi sCMOS camera and 20X 0.75 NA phase contrast air objective, or using an EVOS M7000 automated microscope (Thermo Fisher, UK) using a 10X 0.30 NA objective. Several epifluorescence and transmitted light phase contrast fields-of-view (FOVs) were acquired from each well using the automated stage to cycle through position on the microplate. Cell-associated fluorescence intensity, indicating SNAP-GLP-1R, was analysed by applying a flat-field correction and segmentation of cell-containing regions using Phantast [64] implemented in a macro within Fiji v1.54f (NIH, USA).

### 4.6 β-arrestin recruitment imaging

Stable SNAP-hGLP1R-expressing Flp-In cells, or AD293 cells reverse transfected with SNAP-GLP1R for 24 h using Lipofectamine 2000, were seeded in black-walled, clear-bottomed plates (Ibidi UK) coated with 0.01% poly-d-lysine. 8 h before the assay, mNeonGreen-tagged β-arrestin-1 or β-arrestin-2 BacMam particles (5 μL per well) were added. Cells were labelled with SNAP-Surface AlexaFluor-647 (500 nM) and, in some cases, Hoechst 33342 dye (1:10000; Thermo Fisher, UK) for 10 min at 37 °C, before washing three times with PBS. For live cell imaging, cells were placed in pre-warmed KRBH buffer containing 6 mM glucose, and imaged in a custom built heated enclosure (MicroscopeHeaters.com, UK) at 37 °C before and after addition of 1 μM agonist using the same microscope as in Section 4.5 but using a laser light source (LDI-7, 89North, USA), spinning disc (X-Light V2, CrestOptics, Italy), and 100X 1.45 NA oil immersion objective. The appearance of punctate structures was monitored using the ComDet plugin (https://github.com/UU-cellbiology/ComDet) in Fiji, and quantified as change from baseline. For fixed cell imaging, cells were prepared as above and then stimulated with 1 μM GLP-1, ExD3 or ExF1 at 37 °C before fixation for 10 min using 4% paraformaldehyde (PFA). After further washes, cells were imaged as above.

### 4.7 Borealis β-arrestin conformational activation assay

Stable SNAP-hGLP1R-expressing Flp-In cells were seeded in black-walled, clear-bottomed plates (Greiner, UK) coated with 0.01% poly-d-lysine for 24 h 8 h before the assay, BacMam-encoded Borealis β-arrestin-2 sensor particles (5 μL per well) were added. Alternatively, AD293 cells were reverse transfected with SNAP-GLP1R plasmids using Lipofectamine 2000, and Borealis particles (2 μL per well) were added 16 h before the assay. For the assay, cells were labelled with SNAP-Surface AlexaFluor-647 (500 nM) for 10 min at 37 °C, washed three times with PBS, and placed in KRBH buffer with 6 mM glucose for imaging. The assay was performed using the same microscope as in Section 4.5 using a 10X or 20X air objectives, with several FOVs acquired per well. SNAP-GLP1R was imaged at baseline using the Cy5 channel, and the β-arrestin sensor was imaged using the FITC channel at frequent intervals before and after addition of agonist or vehicle. For transiently transfected AD293 cells, a macro implemented in Fiji was used to extract sensor responses specifically from cells with SNAP-GLP1R expression above a consistent intensity threshold; for stable Flp-In cells responses from all Borealis-expressing cells were included as all cells express GLP-1R. A trace from a well without GLP-1R transfection was subtracted to account for sensor photobleaching. Sensor responses were expressed relative to sensor intensity at baseline and quantified using AUC.

### 4.8 β-arrestin GLP-1R-Nluc recruitment BRET assays

AD293 cells were reverse co-transfected with SNAP-GLP1R-Nluc and pcDNA3.1 in a 1:3 ratio using Lipofectamine 2000 in 12-well plates 24 h before the assay, with BacMam-encoded mNeonGreen-tagged β-arrestin-1 or -2 (50 μL per well) added at the time of seeding. Alternatively, β-arrestin-2-YFP was used in place of pcDNA3.1 and no BacMam β-arrestin particles were used. For end-point assays, cells were then detached, pelleted, and resuspended in KRBH buffer containing 6 mM glucose and 0.1% BSA, before transfer to opaque white 96-well plates (Greiner) containing agonist or vehicle and, after the indicated stimulation period at 37 °C, furimazine (1:700; Promega, UK) was added to each well and BRET signal was recorded using a Glomax plate reader (Promega) at 37 °C, with a 460 nm bandpass and 525 nm longpass filter for donor and acceptor, respectively. For kinetic assays, cells were resuspended in KRBH buffer containing 6 mM glucose and 0.1% BSA supplemented with furimazine (1:700) and transferred to opaque white 96-well plates before transfer to the plate reader; BRET signal was then recorded at regular intervals before and after agonist addition. BRET ratio was calculated from the acceptor:donor emission ratio. For kinetic assays, ligand-induced changes were expressed by subtracting baseline and vehicle responses (ΔΔ BRET).

### 4.9 Bystander BRET assays for β-arrestin and Nb37

Stable SNAP-hGLP1R Flp-In cells were reverse co-transfected with β-arrestin-1-Nluc, β-arrestin-2-Nluc or Nb37-Nluc, plus KRAS-Venus, Rab5-Venus or Rab7-Venus, in a 1:5 ratio, in white tissue-culture-treated plates (Greiner) coated with poly-d-lysine. After 24 h, media was removed and replaced with KRBH buffer containing 6 mM glucose, 0.1% BSA and furimazine (1:250), and BRET changes were measured as in Section 4.8. BRET ratio was calculated from the acceptor:donor emission ratio and ligand-induced changes were expressed by subtracting baseline and vehicle responses (ΔΔ BRET).

### 4.10 BRET assay for detection of GLP-1R disappearance from the plasma membrane

AD293 cells were reverse transfected with SNAP-GLP1R-Nluc plus Venus-tagged KRAS or Rab5 markers (1:3 ratio) using Lipofectamine 2000 for 24 h. Cells were detached, pelleted, and resuspended in KRBH buffer containing 6 mM glucose, 0.1% BSA and furimazine (1:700; Promega), before transfer to opaque white 96-well plates (Greiner). BRET signal was recorded before and after agonist addition using a Glomax plate reader and agonist-induced ΔΔ BRET was calculated as in Section 4.8.

### 4.11 TIRF-based measurement of receptor clustering and internalisation in live cells

Stable SNAP-hGLP1R Flp-In cells were seeded in 18-well chambered polymer coverslips (Ibidi) coated with 0.01% poly-d-lysine. Cells were labelled with SNAP-Surface AlexaFluor-647 (500 nM) for 10 min at room temperature. Labelling media was replaced by KRBH containing 6 mM glucose after two washes with PBS. Cells were imaged using a Nanoimager microscope (ONI, UK) in TIRF mode before and at 30 seconds intervals after addition of agonist or vehicle, with time series captured from at least 3 FOV per well. The numbers of cell surface GLP-1R clusters were quantified using the ComDet plugin in Fiji, and expressed as a change from baseline, with the vehicle trace subtracted to isolate agonist-mediated changes. Changes in fluorescence intensity over time, reflecting disappearance of GLP-1R from the cell surface, were determined from cells thresholded from a maximum intensity projection, and expressed relative to baseline. Background signal from non-cell areas subtracted; the vehicle trace was also subtracted to isolate agonist-mediated changes and to account for photobleaching.

### 4.12 TIRF-based measurement of internalisation in fixed cells

Stable SNAP-hGLP1R Flp-In cells were seeded in 18-well chambered polymer coverslips (Ibidi) coated with 0.01% poly-d-lysine. Cells were labelled with SNAP-Surface AlexaFluor-647 (500 nM) for 10 min at room temperature. Agonist was then added without removing labelling medium, aiming to allow new GLP-1R appearing at the plasma membrane to be labelled. After a 30-minute stimulation at 37 °C, cells were washed twice in PBS and fixed with 4% paraformaldehyde (PFA) for 10 min. After a final wash, cells were imaged using a Nanoimager microscope in TIRF and epifluorescence mode by rapid tile scanning to capture 1–2 mm area per well. SNAP-GLP-1R fluorescence intensity was expressed as TIRF:epifluorescence ratio after first subtracting a fixed background signal, and then the agonist values were expressed as a percentage change from vehicle.

### 4.13 Live cell endosomal puncta formation

Stable SNAP-hGLP1R Flp-In cells seeded in black-walled, clear-bottomed plates (Ibidi, UK) coated with 0.01% poly-d-lysine. Cells were labelled with SNAP-Surface AlexaFluor 647 (500 nM) for 10 min at 37 °C, washed twice with PBS, placed in KRBH buffer containing 6 mM glucose, and imaged in the same microscope as in Section 4.5 at 37 °C before and after addition of 1 μM agonist using a 40X 0.90 NA air objective. 9 FOVs were acquired per well. Endosomal puncta was detected using the ComDet plugin in Fiji, and changes over time expressed relative to the baseline.

### 4.14 High resolution imaging of receptor internalisation in fixed cells

Stable SNAP-hGLP1R Flp-In cells, or wild-type or β-arrestin-1/2 knockout AD293 cells reverse transfected with SNAP-GLP1R for 24 h using Lipofectamine 2000, were seeded in black-walled, clear-bottomed plates (Ibidi) coated with 0.01% poly-d-lysine. Cells were labelled with SNAP-Surface AlexaFluor-647 (500 nM) and Hoechst dye (1:10000) for 10 min at 37 °C, and, after washing three times with PBS, stimulated with 1 μM GLP-1, ExD3 or ExF1 for 30 min at 37 °C before fixation for 10 min using 4% PFA. After further washes, cells were imaged as in Section 4.6. Images were analysed using Fiji.

### 4.15 Measurement of GLP-1R internalisation by reversible SNAP-labelling

Stable SNAP-hGLP1R Flp-In cells, or AD293 cells reverse transfected with SNAP-GLP1R for 24 h using Lipofectamine 2000, were seeded in black-walled, clear-bottomed plates (Greiner) coated with 0.01% poly-d-lysine. Cells were labelled with the cleavable SNAP-tag labelling probe BG–SS–AF647 (500 nM, Biosynth, UK) for 10 min at 37 °C, washed once with PBS, and treated with agonist in serum-free medium containing 0.1% BSA for 30 min at 37 °C to induce GLP-1R internalisation. Cells were then washed twice with PBS and placed in Tris/NaCl/EDTA (TNE) buffer, pH 8.6 for imaging. Cells were then imaged before and after addition of the cell-impermeable reducing agent sodium 2-mercaptoethanesulfonate (Mesna; 100 mM) using the same microscopes as in Section 4.5, using LED and brightfield illumination via a 20X phase contrast air objective. Several epifluorescence and transmitted light phase contrast fields-of-view (FOVs) were acquired from each well using the automated stage to cycle through position on the microplate. Cell-associated fluorescence intensity, indicating internalised GLP-1R, was analysed by applying a flat-field correction and segmentation of cell-containing regions using Phantast. Percentage GLP-1R internalisation was quantified from the pre- and post-Mesna fluorescence, representing total and internalised GLP-1R, respectively.

### 4.16 Colocalisation of internalised GLP-1R with endosomal subpopulations

Stable SNAP-hGLP1R Flp-In cells were reverse transfected with Rab5-Venus, Rab7-Venus or Rab11-Venus for 24 h using Lipofectamine 2000 in black-walled, clear-bottomed plates (Ibidi, UK) coated with 0.01% poly-d-lysine. Cells were pre-labelled with BG–SS–AF647 (500 nM) in serum-free medium containing 0.1% BSA for 10 min at room temperature, after which vehicle or agonist was added without removal of the labelling media, aiming to ensure that any new GLP-1R reaching the plasma membrane during the stimulation phase could be labelled prior to internalisation. Agonists were added in reverse time order and stimulation performed at 37 °C. At the end of the stimulation, labelling media was removed, cells washed once with PBS, and residual surface SNAP-labelling was removed using Mesna (100 mM in TNE buffer, pH 8.6) for 5 min, followed by a further wash in PBS, fixation with 4% PFA for 10 min, and a final wash in PBS. Cells were imaged using an automated Olympus IX83 microscope with a 40X 0.95 NA air objective, Yokogawa CSU-W1 spinning disc unit and Hamamatsu ORCA-Fusion sCMOS camera, controlled by ScanR acquisition software (Evident, UK). 9 FOVs were acquired per well including Z-stacks with 12 planes separated by 1 μm to capture the volume of the cell. Object-based cocolocalisation of Venus- and Cy5-marked endosomal puncta was performed using the ComDet plugin in Fiji. The integrated density of SNAP-GLP1R from each colocalised endosome was extracted from the plugin readout and averaged as an indicator of the amount of GLP-1R in each endosomal compartment. Approximately 300,000 endosomes were analysed per condition within each experiment.

### 4.17 Imaging of Gα_s_ activation

Stable SNAP-hGLP1R Flp-In cells were reverse transfected with Nb37-GFP for a relatively short period (12 h) to reduce overexpression using Lipofectamine 2000 in 18-well chambered polymer coverslips (Ibidi) coated with 0.01% poly-d-lysine. Cells were labelled with SNAP-Surface AlexaFluor-647 (500 nM) for 10 min at room temperature. Labelling media was replaced by KRBH containing 6 mM glucose after two washes with PBS. Cells were imaged using a Nanoimager microscope in TIRF mode before and at 30 second intervals after addition of agonist or vehicle. Changes in Nb37-GFP at the plasma membrane over time were quantified from cells thresholded from a maximum intensity projection and expressed relative to baseline, with background signal from non-cell areas subtracted; the vehicle trace was also subtracted to isolate agonist-mediated changes and to account for photobleaching. To image endosomal Nb37-GFP, cells were treated for 60 min prior to fixation with 4% PFA and imaged in HILO mode.

### 4.18 cAMP HTRF assay

AD293 cells were reverse co-transfected with SNAP-GLP1R plasmids using Lipofectamine 2000 in 12-well plates. Cells were then detached, pelleted and resuspended in serum-free DMEM containing 0.1% BSA and then stimulated for the indicated time at 37 °C with agonist. cAMP was measured by HTRF (cAMP Dynamic kit, Cisbio, France).

### 4.19 Kinome assay

AD293 cells were transiently transfected for 16 h before the assay in 6-well plates. Transfected cells were then stimulated in complete medium with 1 μM agonist or vehicle for 5 or 60 min. After stimulation, the cells were gently washed and stored at −80 °C before shipping to the central PamGene facility (Netherlands) for analysis. Briefly, cell lysates were applied to STK PamChip arrays in a PamStation device, which detects phosphorylation of a panel of target peptide substrates in real time through fluorescence changes. A standard QC and batch-effect correction pipeline was applied to determine phosphorylation signal for each peptide substrate. Agonist-mediated effects were then calculated as log_2_ fold changes relative to the vehicle. ANOVA testing with Dunnett's correction was performed to identify statistically significantly increased changes. Upstream kinase analysis (UKA) was applied to phosphosite data to predict kinase activity using a functional class scoring method based on public databases of kinase-protein-phosphosite relationships. UKA was used to ascribe both a “kinase change”, which represents the direction and magnitude of all phosphosites engaged by that kinase, and a “kinase score”, which represents the significance of the change and its specificity for that particular kinase. All kinases with a kinase score >1.3 were included for downstream analysis.

### 4.20 Pancreatic islet isolation

Mouse pancreas isolation was carried out by collagenase digestion. Collagenase (1 mg/mL) was dissolved in RPMI-1640 and kept on ice. Islet isolation was performed immediately after cervical dislocation under a microscope. The pancreas was inflated via the common bile duct, and density separation was carried out by layering Histopaque 1119 and 1083. Islets were handpicked under a microscope and cultured at 37 °C with 5% CO_2_ in RPMI supplemented with 10% FBS, 1% penicillin-streptomycin.

### 4.21 Imaging of adenovirally expressed GLP-1R distribution in pancreatic islets

After isolation, *Glp1r* KO islets were transduced with adenoviral particles (2.5∗10^8^ per 200 islets) encoding untagged hGLP1R^WT^ or hGLP1R^TM^. 20 h later, islets were batch treated in complete RPMI-1640 with 100 nM ExD3-Cy5 or ExF1-Cy5 for 30 min, before washing twice in KRBH. Washed islets were transferred to an Ibidi 8-well imaging chamber in KRBH and imaged using the same microscope as in Section 4.5 in spinning disc confocal mode using a 40X 1.30 NA oil immersion objective. Multiple islets were imaged per acquisition.

### 4.22 cADDis cAMP measurements in adenovirally transduced pancreatic islets

After isolation, *Glp1r* KO islets were dispersed by trituration for 3 min in EDTA/trypsin at 37 °C. Dispersed islet cells were transduced with cADDis BacMam viral particles and seeded onto black, clear-bottomed 96-well plates pre-coated with 0.01% poly-d-lysine and 25 μg/mL mouse laminin (Thermo Fisher) for 24 h. Untagged hGLP1R^WT^ or hGLP1R™ adenoviral particles (0.5∗10^7^ per well) were also co-transduced. For imaging, media was removed and replaced with KRBH buffer containing 6 mM glucose and 0.1% BSA and cells pre-incubated for 30 min at 37 °C. Imaging was performed using the same microscope as in Section 4.5, with several fields-of-view per well imaged before and after 100 nM agonist or vehicle addition. At the end of the incubation, IBMX (500 μM) and forskolin (50 μM) were added to stimulate a maximal sensor response. A macro implemented in Fiji was used to extract signal kinetics, based on a drift-corrected image stack with segmentation of cells according to maximum intensity projection. Sensor changes were normalised to the maximum response to IBMX/forskolin and to baseline, depending on the specific analysis required. Cells which failed to show an IBMX/forskolin response greater than a 1.5-fold increase to baseline were excluded. Averaged data from hundreds of cells imaged in parallel were used for each biological replicate.

### 4.23 Insulin secretion

INS-1 cells with knockout of endogenous GLP-1R were seeded in 96-well clear bottom, tissue-culture treated plates and transfected with hGLP1R plasmids for 24 h in complete RPMI-1640 medium at 11 mM glucose. For the final 16 h, 100 nM agonist (100 nM) or vehicle were added. At the end of the incubation, a sample of supernatant was collected and analysed for insulin concentration by HTRF (Insulin High Range HTRF kit, Cisbio). Results were expressed relative to that obtained using untransfected cells (i.e. no GLP-1R) tested in parallel.

### 4.24 Statistical analysis

For all experiments, independent biological replicates were considered as the average of technical replicates from each experiment. Matched designs, i.e. with all treatments performed in parallel, were used wherever possible. Mouse pancreatic islet experiments were performed without regard to sex, with both sexes used. Statistical comparisons were performed using Prism 10.5.1 (GraphPad Software) using the tests described in the figure legend. For concentration-response analyses three-parameter logistic fitting was performed using Prism. Scaling to experimental averages was performed to reduce the impact of irrelevant differences in numerical data e.g. related to microscope intensity settings. Statistical significance was inferred when p < 0.05, with additional levels of significance as described in the figure legends. Data are represented as mean ± SEM, with individual experimental replicates were possible.

## CRediT authorship contribution statement

**Hanh Duyen Tran:** Writing – review & editing, Investigation, Formal analysis. **Yiming Zuo:** Writing – review & editing, Investigation, Formal analysis. **Carissa Wong:** Writing – review & editing, Investigation. **Alice Pollard:** Writing – review & editing, Investigation. **Steve Bloom:** Writing – review & editing, Supervision, Funding acquisition. **Ben Jones:** Writing – original draft, Supervision, Project administration, Methodology, Investigation, Funding acquisition, Formal analysis, Data curation, Conceptualization.

## Funding

B.J. acknowledges funding from the Medical Research Council (MR/Y00132X/1 and MR/X021467/1), the Wellcome Trust (301619/Z/23/Z), and Metsera Inc. A.P. is supported by a BBSRC Discovery Fellowship (BB/W009633/1). The Section of Endocrinology and Investigative Medicine at Imperial College London is funded by grants from the MRC, NIHR and is supported by the NIHR Biomedical Research Centre Funding Scheme and the NIHR/Imperial Clinical Research Facility.

## Declaration of competing interest

Ben Jones has received funding from Eli Lilly and Metsera Inc, and acts as a consultant for Metsera Inc. Steve Bloom is an employee of and shareholder in Metsera Inc and Ben Jones and Steve Bloom have received research funding from Metsera Inc, which is developing gut hormone analogues for treatment of metabolic disease.

## Data Availability

Data will be made available on request.
