# Supplementary material for: Modelling G protein-biased agonism using GLP-1 receptor C-terminal mutations
Source: Mol Metab. 2026 Jan 20;105:102321. doi: 10.1016/j.molmet.2026.102321 (PMC12925471; doi:10.1016/j.molmet.2026.102321)
Supplement: Multimedia component 3 [file mmc3.pdf]

Supplementary Figures

Modelling G protein-biased agonism using GLP-1 receptor C-terminal mutations

Hanh Duyen Tran[1,*], Yiming Zuo[1,*], Carissa Wong[1], Alice Pollard[2], Steve Bloom[1], Ben Jones[1,#].

[1] Section of Endocrinology, Department of Metabolism, Digestion and Reproduction, Faculty of Medicine, Imperial College London, Du Cane Road, W12 0NN, United Kingdom.
[2] Institute of Clinical Sciences, Faculty of Medicine, Imperial College London, Du Cane Road, W12 0NN, United Kingdom.

A

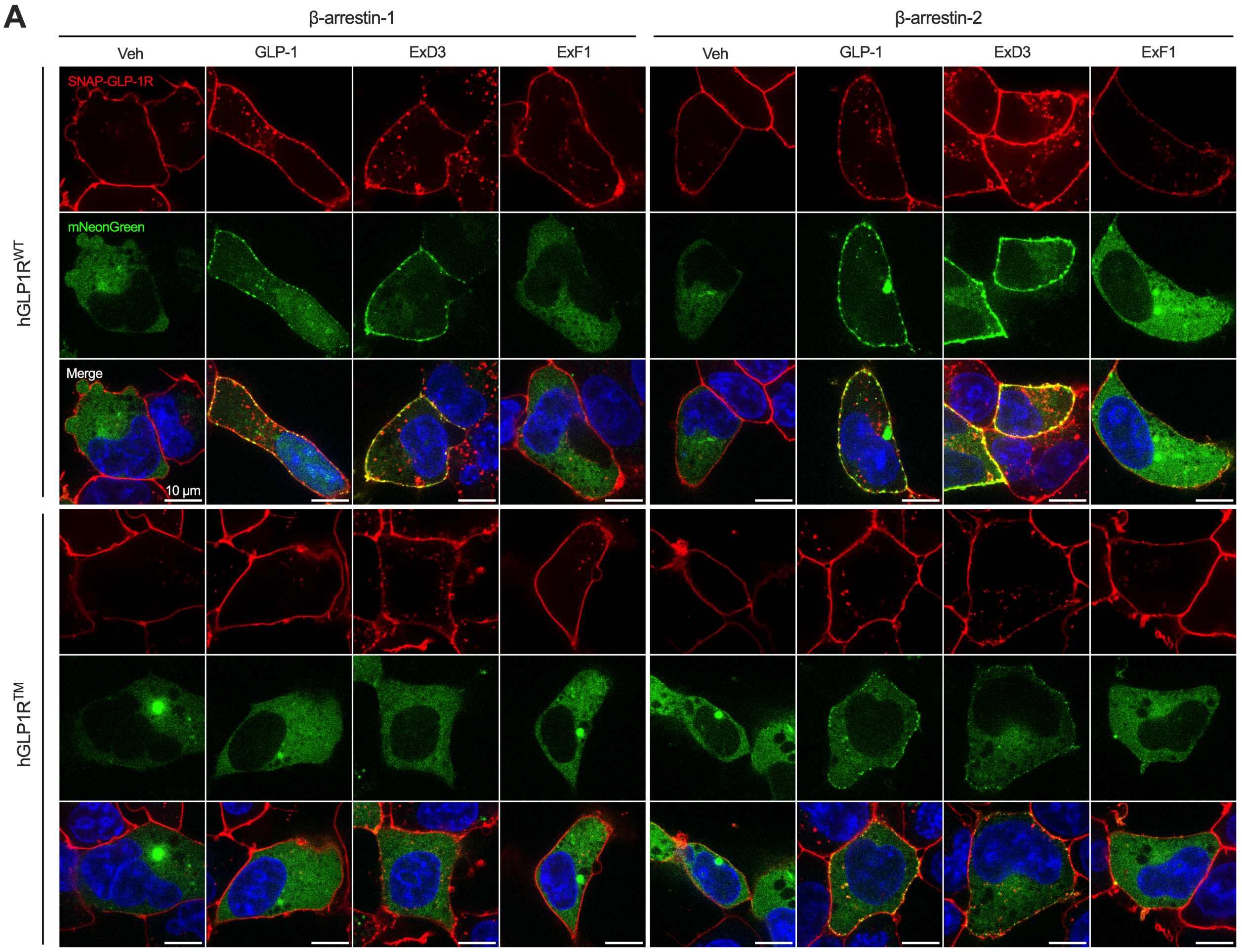

B

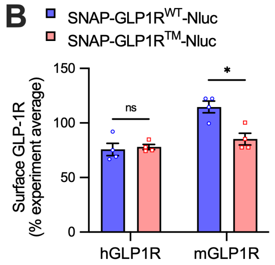

C

	β-arrestin-1															
	**hGLP1R-WT**				**hGLP1R-TM**				**mGLP1R-WT**				**mGLP1R-TM**			
	Emax		LogEC50		Emax		LogEC50		Emax		LogEC50		Emax		LogEC50	
	Mean	SEM	Mean	SEM	Mean	SEM	Mean	SEM	Mean	SEM	Mean	SEM	Mean	SEM	Mean	SEM
GLP-1	0.0176	0.0022	-7.06	0.21	0.0039	0.0014	-7.48	0.72	0.0185	0.0016	-7.39	0.18	0.0071	0.0012	-7.56	0.37
ExD3	0.0167	0.0015	-7.82	0.20	0.0043	0.0009	-8.20	0.52	0.0179	0.0011	-8.48	0.16	0.0073	0.0009	-9.07	0.35
	**β-arrestin-2**															
	hGLP1R-WT				**hGLP1R-TM**				**mGLP1R-WT**				**mGLP1R-TM**			
	Emax		LogEC50		Emax		LogEC50		Emax		LogEC50		Emax		LogEC50	
	Mean	SEM	Mean	SEM	Mean	SEM	Mean	SEM	Mean	SEM	Mean	SEM	Mean	SEM	Mean	SEM
GLP-1	0.0246	0.0027	-6.68	0.16	0.0079	0.0011	-7.22	0.25	0.0167	0.0012	-7.11	0.12	0.0107	0.0021	-6.72	0.29
ExD3	0.0218	0.0018	-7.54	0.17	0.0102	0.0009	-7.74	0.20	0.0154	0.0010	-8.13	0.15	0.0089	0.0010	-7.78	0.25

D

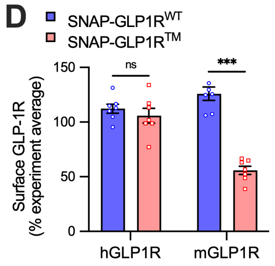

E

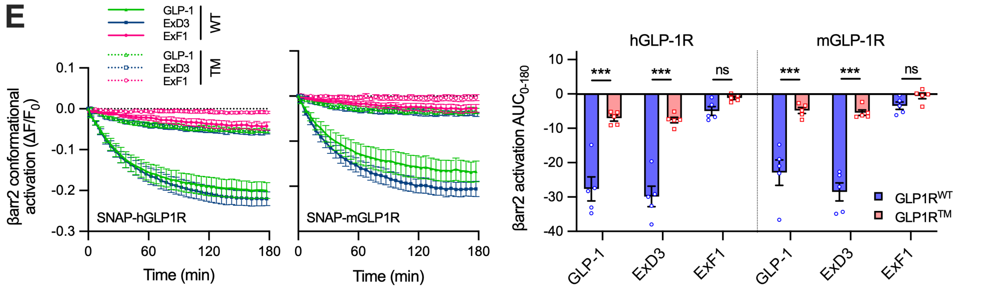

Supplementary Figure 1. (**A**) Representative images from *n*=3 experiments showing stable SNAP-hGLP1R^{WT} and SNAP-hGLP1R^{WT} cells transduced with mNeonGreen-tagged β-arrestin-1 or -2, 5 minutes after stimulation with vehicle or 1 µM agonist, scale bar = 10 µm. (**B**) Surface expression quantification of C-terminal nanoluciferase tagged SNAP-hGLP1R^{WT} and SNAP-hGLP1R^{WT} transiently expressed in AD293 cells, *n*=4, two-way matched ANOVA with Sidak test. (**C**) E_{max} and $LogEC_{50}$ values from 3-parameter logistic fitting of pooled data shown in Fig 1F and 1G. Note, fitting of ExF1 responses was not reliable. (**D**) Surface expression quantification of SNAP-hGLP1R^{WT} and SNAP-hGLP1R^{WT} (no luciferase tag) transiently expressed in AD293 cells, *n*=7, two-way matched

ANOVA with Sidak test. (**E**) β-arrestin-2 Borealis conformational activation in transiently transfected AD293 cells in response to 1 μM agonist, *n*=5, with comparison of AUC by two-way matched ANOVA with Sidak's test. *$p<0.05$, ***$p<0.001$ by indicated statistical test. All data shown as mean ± SEM, with individual replicates in some cases.

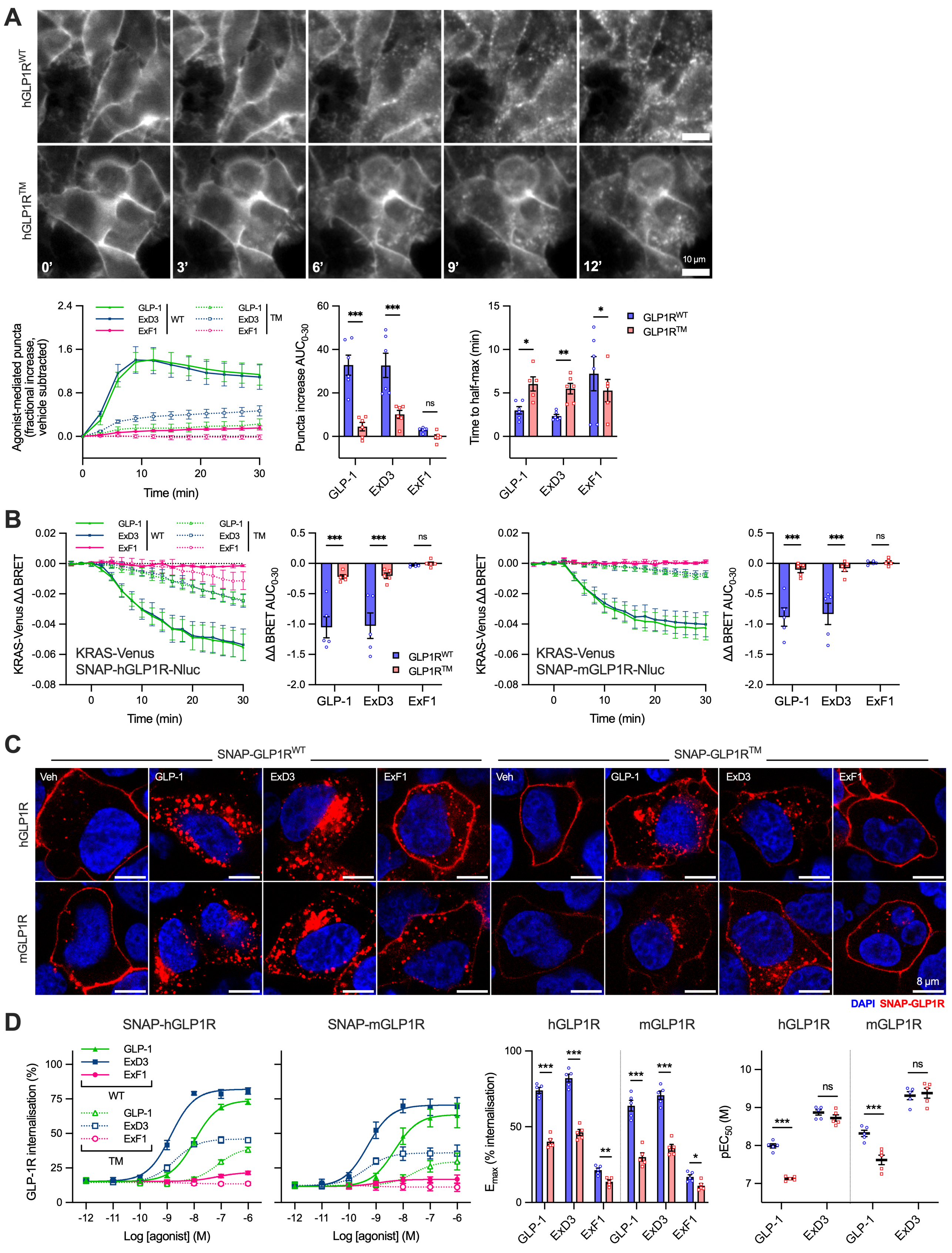

Supplementary Figure 2. (**A**) GLP-1R internalisation kinetics in stable SNAP-hGLP1R^{WT} or SNAP-hGLP1R^{TM} cells quantified by appearance of endosomal puncta, *n*=6, with comparisons of both AUC and time to half-max (one phase association fit) by two-way matched ANOVA with Sidak's test. Scale bar = 10 µm. (**B**) GLP-1R internalisation measured via BRET between transiently transfected SNAP-

GLP1R-Nluc and KRAS-Venus in AD293 cells, *n*=5, with comparisons between AUC by two-way matched ANOVA with Sidak's test. (**C**) Representative images showing AD293 cells expressing human or mouse SNAP-GLP1R^{WT} or SNAP-GLP1R^{TM} 30 minutes after stimulation with vehicle or 1 μM agonist, scale bar = 8 μm. (**D**) GLP-1R internalisation at 30 minutes in transiently transfected AD293 cells quantified by reversible SNAP-GLP1R labelling, n=5, with statistical comparisons between E_{max} and pEC_{50} by two-way matched ANOVA with Sidak's testm *p<0.05, **p<0.01, ***p<0.001. All data shown as mean ± SEM, with individual replicates in some cases.

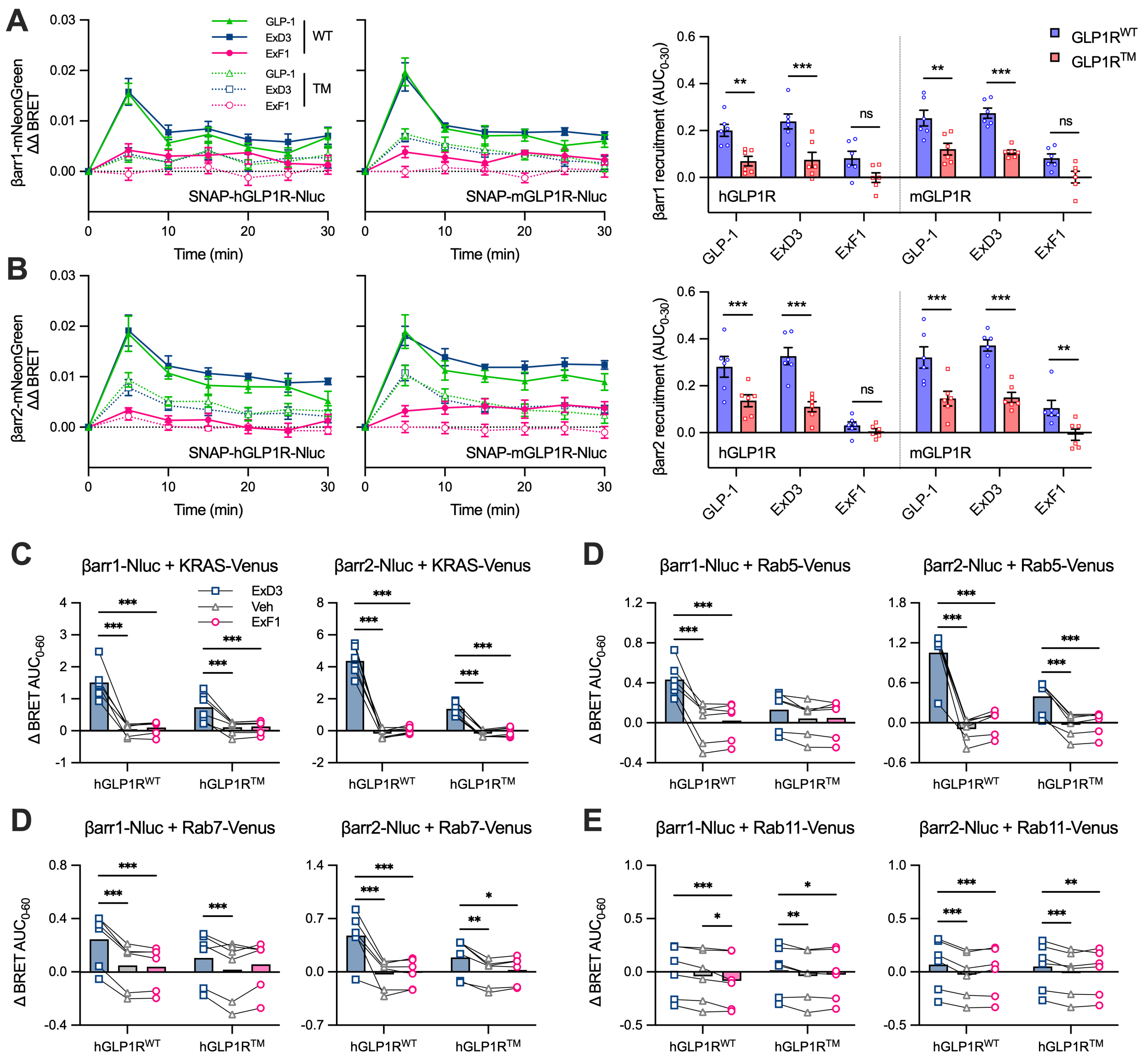

Supplementary Figure 3. (**A**) β-arrestin-1-mNeonGreen recruitment to SNAP-GLP-1R-Nluc in transiently transfected AD293 cells over 30 minutes with GLP-1, ExD3 or ExF1, n=6, AUCs compared by two-way matched ANOVA with Sidak's test. (**B**) As for (A) but β-arrestin-2-mNeonGreen. (**C**) to (**E**) show AUC analysis of data from Fig 3B and 3C, i.e. recruitment of β-arrestin-1- or -2-Nluc to plasma membrane (KRAS-Venus), early endosomes (Rab5-Venus), late endosomes (Rab7-Venus) or recycling endosomes (Rab11-Venus) over 60 minutes, but without vehicle subtraction. Statistical analysis is by two-way matched ANOVA with Tukey's test. *p<0.05, **p<0.01, ***p<0.001 by indicated statistical test. All data shown as mean ± SEM, with individual replicates in some cases.

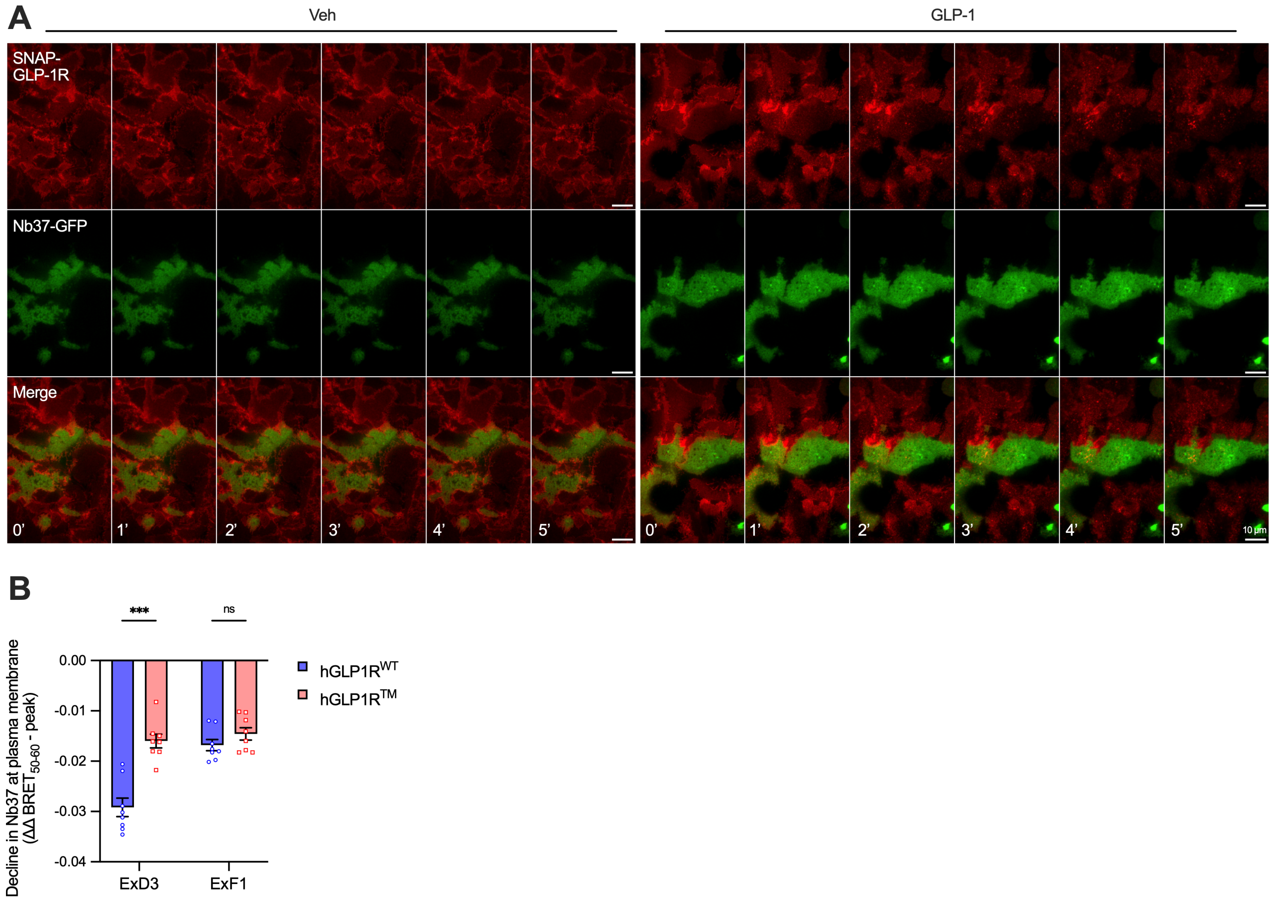

Supplementary Figure 4. (**A**) Additional images showing full field-of-view of 1 µM GLP-1- or vehicle-induced Nb37-GFP plasma membrane recruitment in SNAP-hGLP1R^{WT} cells, scale bar = 10 µm. (**B**) Comparison in the magnitude of the decline in plasma membrane Gα_s activation over time, calculated from data shown in Fig 4C as peak Nb37-Nluc/KRAS-Venus ΔΔBRET signal minus the mean value from the final 10 minutes of the stimulation. Statistical comparison by two-way matched ANOVA with Sidak's test, ***p<0.001. Data shown as mean ± SEM with individual replicates.

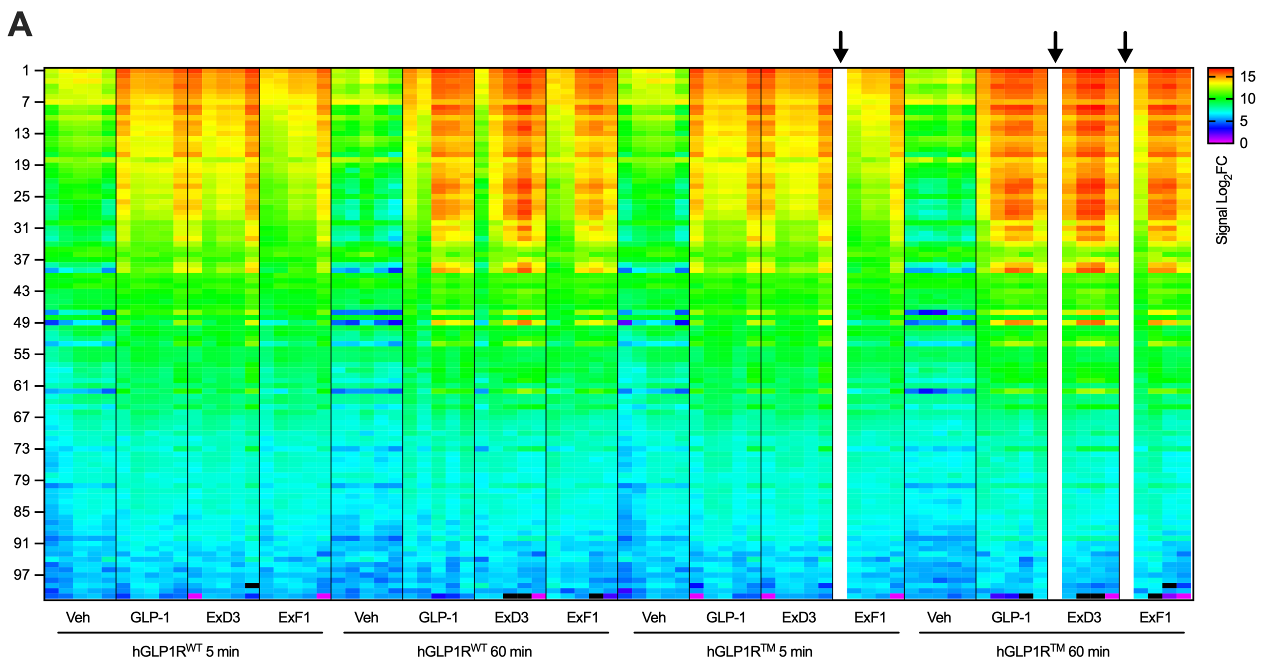

Supplementary Figure 5. (**A**) Substrate phosphorylation for each sample used in the kinome array. Note that 3 samples (arrows) showed no kinase activity and were excluded.

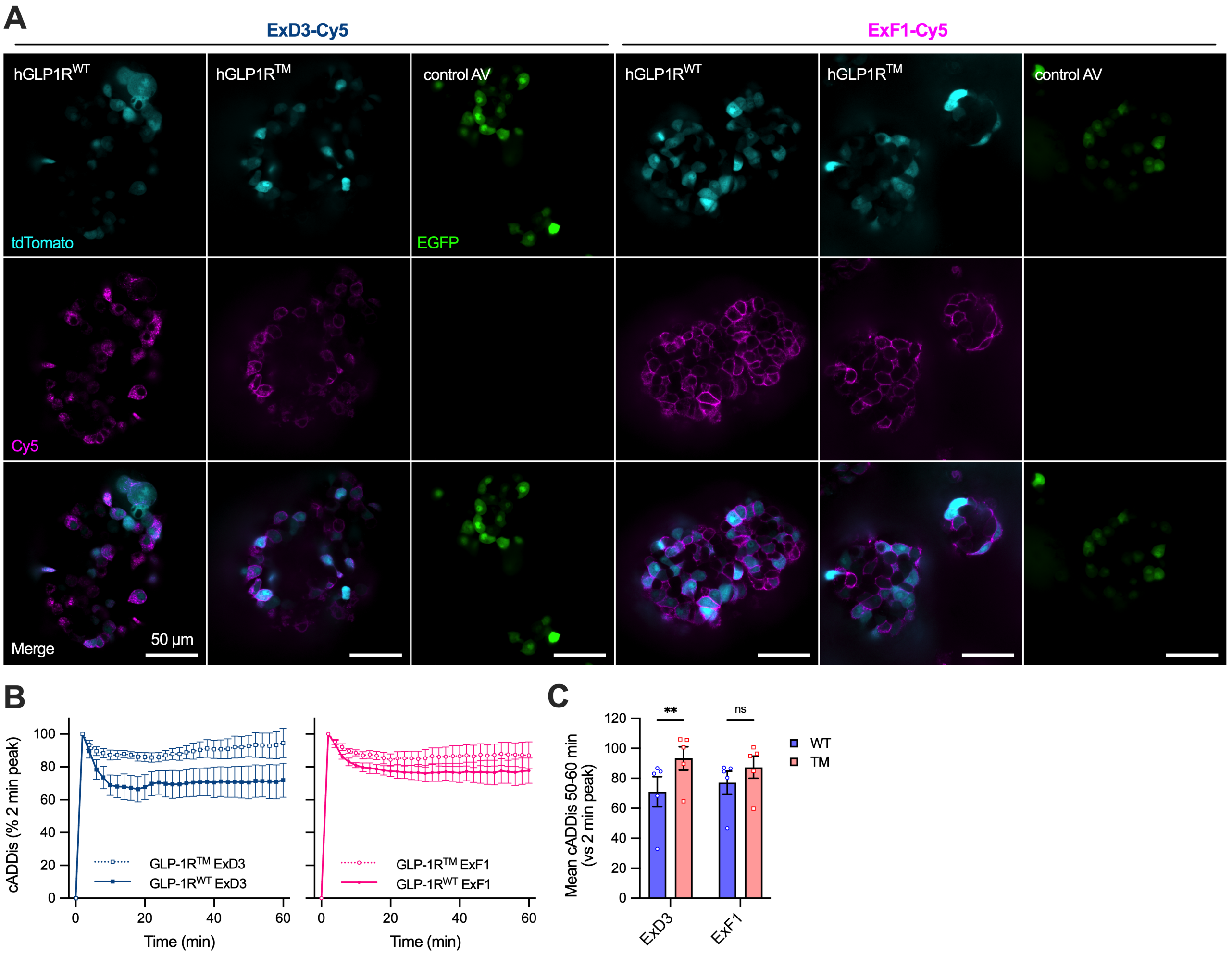

Supplementary Figure 6. (**A**) Related to Fig 6A: confocal images of *Glp1r* KO islets transduced with hGLP1R^{WT} or hGLP1R^{TM} adenovirus and treated with 100 nM ExD3-Cy5 or ExF1-Cy5 for 30 minutes before imaging; the transduction control fluorescent proteins (EGFP or tdTomato) are shown. Scale bar = 50 µm. (**B**) Alternative analysis of data from Fig 6D showing cADDis cAMP responses to 100 nM agonist, n=5, with normalisation to the peak measured at 2 minutes. (**C**) Quantitative comparison of data from (B), with final 10-minute mean signal expressed relative to the peak measured at 2 minutes, with two-way matched ANOVA with Sidak's test.

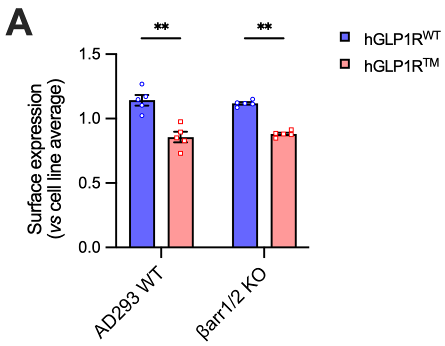

Supplementary Figure 7. (**A**) Surface SNAP-GLP1R labelling, *n*=5, with comparison by two-way matched ANOVA with Sidak's test; **p<0.05. All data shown as mean ± SEM, with individual replicates in some cases.

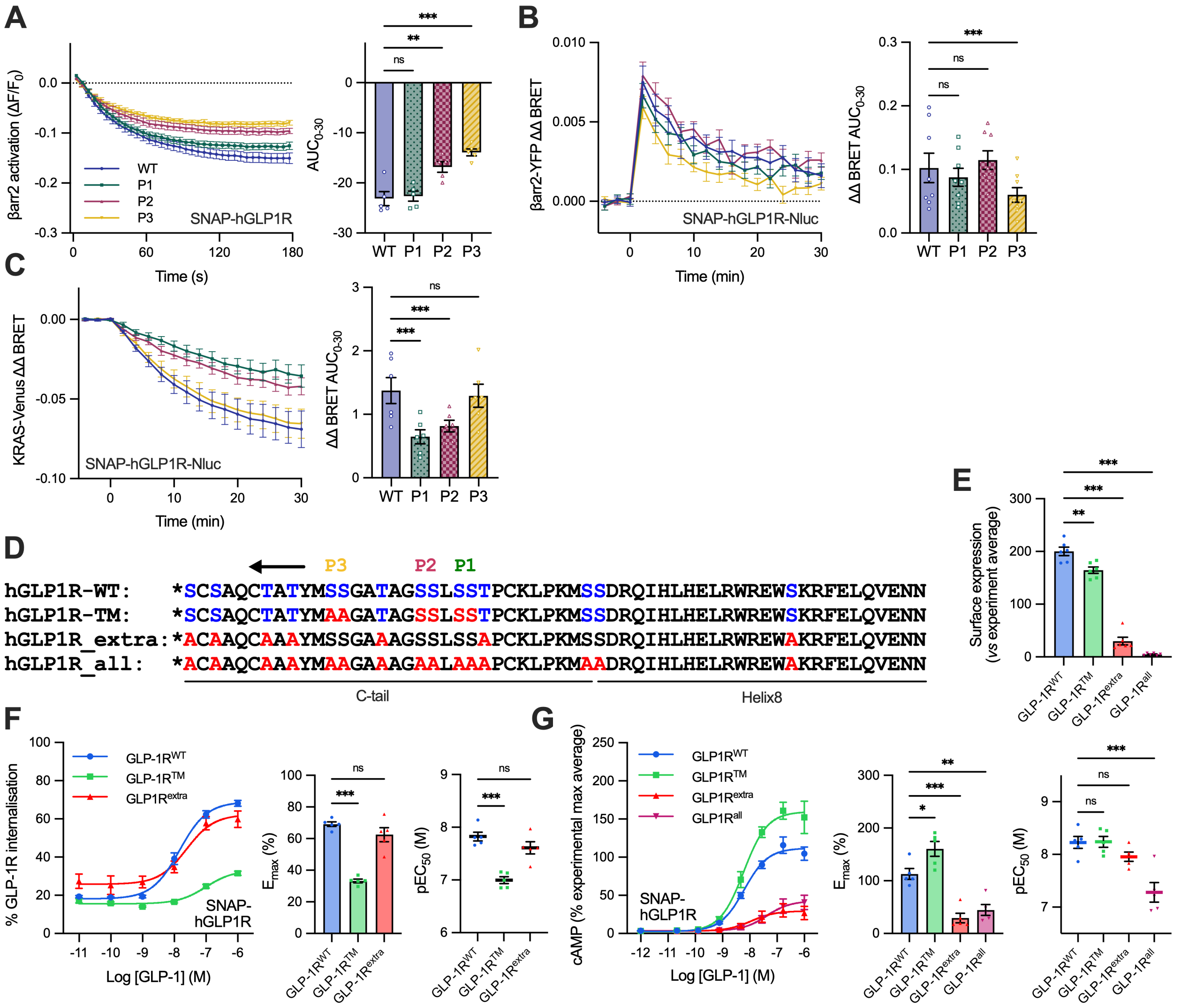

Supplementary Figure 8. (**A**) Borealis β-arrestin-2 conformational activation in AD293 cells expressing SNAP-GLP1R constructs, *n*=5; AUC with comparison by one-way matched ANOVA with Dunnett's test. (**B**) β-arrestin-2-YFP recruitment time-course in AD293 cells expressing SNAP-GLP1R-Nluc constructs, *n*=8, AUC with comparison by one-way matched ANOVA with Dunnett's test. (**C**) GLP-1R internalisation measured via BRET between SNAP-GLP1R-Nluc and KRAS-Venus in AD293 cells, *n*=6, AUC with comparisons by one-way matched ANOVA with Dunnett's test. (**D**) hGLP1R^{extra} and hGLP1R^{all} C-tail sequences aligned to the triple mutant and wild-type sequences. (**E**) Surface expression using SNAP-tag labelling in AD293 cells, *n*=6 for SNAP-GLP1R and one-way matched ANOVA and Dunnett's test comparing mutation effects. (**F**) GLP-1-induced GLP-1R internalisation at 30 minutes in AD293 cells measured via reversible SNAP-GLP1R labelling, *n*=5, E_{max} and pEC_{50} compared by one-way matched ANOVA with Dunnett's test. (**G**) cAMP accumulation in AD293 cells measured at 30 minutes, *n*=5, E_{max} and pEC_{50} compared by one-way matched ANOVA with Dunnett's test. *p<0.05, **p<0.01, ***p<0.001 by indicated statistical test. All data shown as mean ± SEM, with individual replicates in some cases.
